# DCPR: a deep learning framework for circadian phase reconstruction

**DOI:** 10.1186/s12859-025-06363-2

**Published:** 2025-12-30

**Authors:** Xiao Han, Xiaochen Cen, Zhijin Li, Xiaobo Zhou, Zhiwei Ji

**Affiliations:** 1 College of Artificial Intelligence, Nanjing Agricultural University, No. 666 Binjiang Avenue, Nanjing, 211800 Jiangsu China; 2 Center for Data Science and Intelligent Computing, Nanjing Agricultural University, No. 666 Binjiang Avenue, Nanjing, 211800 Jiangsu China; 3 Department of Neurosurgery, Division of Life Science and Medicine, The First Affiliated Hospital of USTC (Anhui Provincial Hospital), University of Science and Technology of China, Hefei, 230036 Anhui China; 4 School of Biomedical Informatics, The University of Texas Health Science Center at Houston, 7000 Fannin Street, Houston, TX 77030 USA

**Keywords:** Circadian rhythm, Gene expression, Circadian variation, Phase reconstruction, Alzheimer’s disease

## Abstract

**Background:**

The circadian clock is an evolutionarily conserved system that orchestrates 24-h physiological rhythms through transcriptional and translational feedback loops. Mounting evidence suggests a bidirectional relationship between circadian rhythm alteration and disease progression, positioning the circadian clock as a potential therapeutic target. Due to the scarcity of high-resolution temporal omics data, it remains very challenging to elucidate the underlying regulatory mechanisms of the circadian system. As a practical alternative, public untimed transcriptomic datasets offer the potential to infer gene expression oscillations retrospectively. However, existing computational approaches for circadian phase estimation often suffer from limited predictive accuracy, reducing their ability to reliably reconstruct rhythmic gene expression patterns.

**Results:**

To overcome these limitations, we develop DCPR, an unsupervised deep learning framework designed to accurately reconstruct the circadian phase from untimed transcriptomic data. Through comprehensive analyses of both simulated and real data, DCPR consistently overperforms existing methods in circadian phase estimation. Additional validations using knowledgebase mining and *ex vivo* experimental data further support DCPR’s efficacy in reconstructing the oscillatory pattern of gene expression and detecting circadian variation.

**Conclusions:**

Our study demonstrates that DCPR is a highly versatile tool for systematically identifying transcriptional rhythms from untimed expression data. This tool will facilitate therapeutics discovery for circadian-related behavioral and pathological disorders.

**Supplementary Information:**

The online version contains supplementary material available at 10.1186/s12859-025-06363-2.

## Background

The circadian clock is an evolutionarily conserved biological system that enables organisms, from cyanobacteria to mammals, to anticipate and adapt to daily environmental fluctuations [1, 2]. This system governs 24-h physiological rhythms, such as the sleep-wake cycles, through cell-autonomous molecular oscillators comprising core clock genes [3]. These genes participate in transcriptional-translational feedback loops that drive the rhythmic expression of clock-controlled genes (CCGs), thereby regulating a wide range of cellular and systemic functions [4, 5]. Circadian disruption is increasingly recognized not merely as a risk factor but as a potential driver of various pathologies, including neurological disorders and cancer [6, 7]. The growing evidence highlights the clinical relevance of circadian biology and its potential as a target for therapeutic intervention [8–13].

Elucidating the molecular basis of circadian rhythms requires a comprehensive analysis of gene expression oscillatory patterns and their alteration in disease contexts [14, 15]. Traditional approaches typically rely on time-series transcriptomic data analyzed using the non-parametric method (JTK_CYCLE [16]) or cosine-based regression models (*e.g*., Cosinor [17], ARSER [18]). However, these methods face significant practical limitations. Generating high-temporal-resolution data demands tightly controlled environmental conditions, extensive biological replication, and frequent sampling, all of which substantially increase experimental costs and complexity [19–21]. Although public repositories, including GEO, GTEx, EBI, etc., contain vast transcriptional datasets, most of these datasets lack precise timing-of-collection metadata [22]. Therefore, this gap underscores the need for robust, unsupervised algorithms capable of accurately inferring the circadian phase for each sample from untimed public transcriptomic data. Such approaches would enable genome-wide reconstruction of gene expression rhythms, providing a powerful alternative for circadian research.

Existing unsupervised approaches for circadian phase inference, such as CYCLOPS and CHIRAL [22, 23], aim to identify an optimal circular projection in a two-dimensional (2D) space that best captures the structures of the data by maximizing the data likelihood. Unfortunately, a systematic quantitative comparison of their robustness and generalizability across datasets is lacking. In parallel, several algorithms (*e.g*., CCPE and Cyclum, etc.) have been developed to perform similar unsupervised phase inference tasks for identifying cell cycle stages using single-cell RNA sequencing (scRNA-seq) data [24, 25]. These methods typically infer cell cycle stages by clustering cells based on estimated pseudo-time in 2D space [26]. Nevertheless, they focus primarily on overall cell-cycle ordering rather than their precise positioning along the circular trajectory. Consequently, their applicability for estimating the phase of samples remains unclear. For reconstructing periodic patterns of gene expression from small sample datasets, it is particularly crucial to achieve high per-sample prediction accuracy. Thus, developing reliable computational tools to accurately reconstruct circadian patterns of gene expression from existing untimed transcriptomic data remains a significant challenge.

**Fig. 1:**
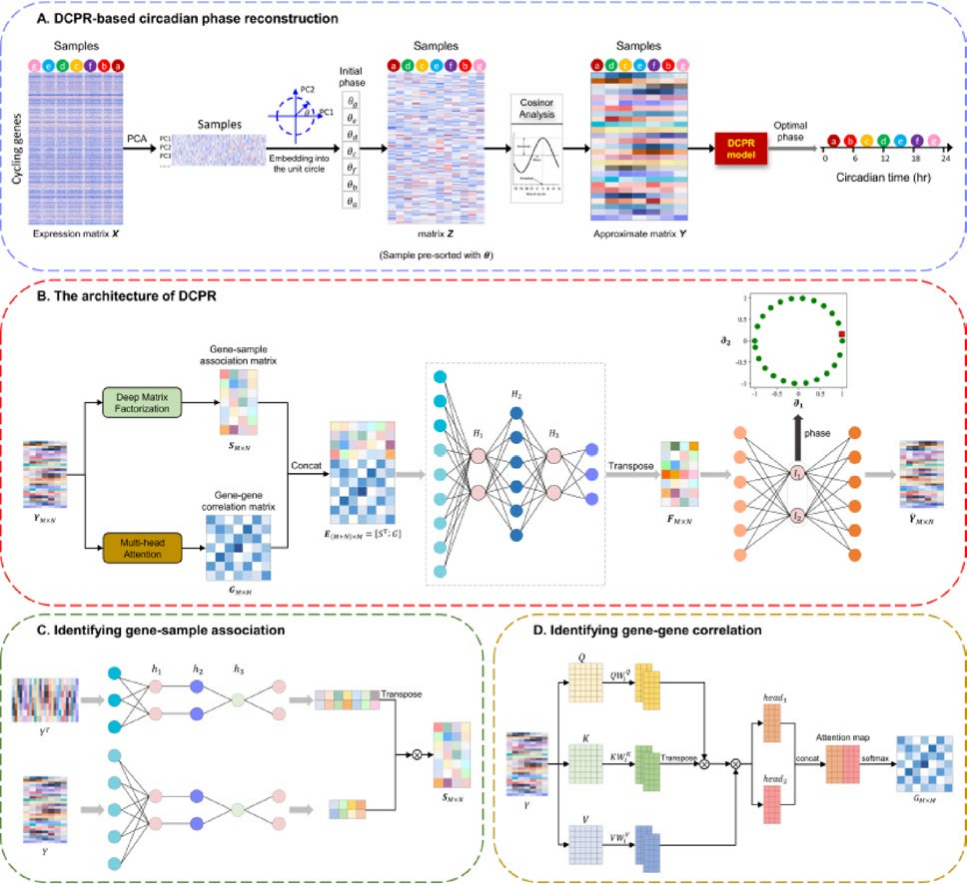
Overview of the DCPR model. **A** the whole procedure of DCPR for phase reconstruction; **B** the architecture of DCPR model; **C** the module for identifying gene-sample association; **D** the module for identifying gene–gene correlation

**Fig. 2:**
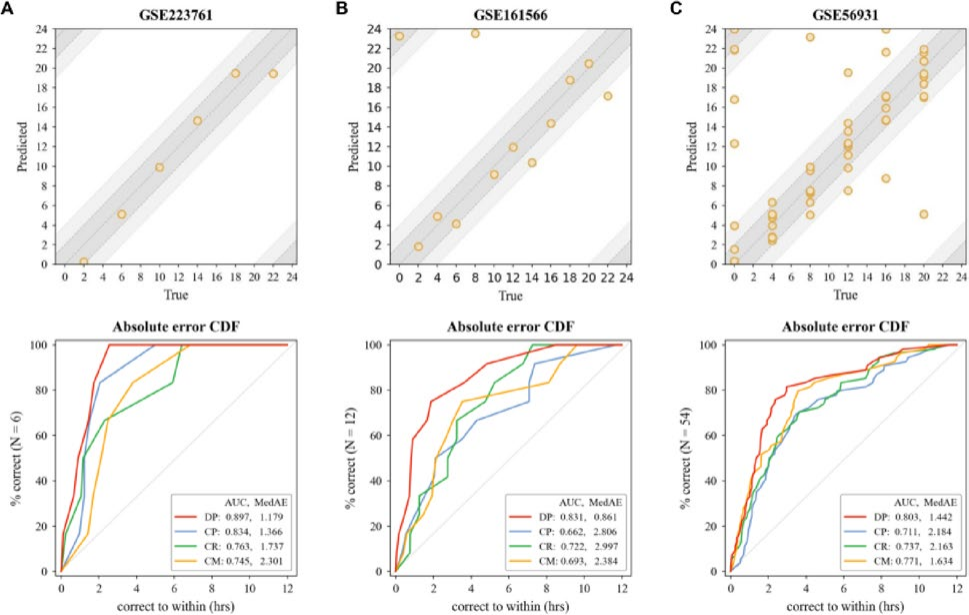
Accuracy of DCPR leveraging evolutionary conservation across three real datasets **A** GSE223761, **B** GSE161566, and **C** GSE56931. Each column corresponds to the sample plot and AUC curve for one dataset. In the top row, the predicted time of day vs. true time of day for each sample is shown. Dark and light gray bands indicate an error range of $\pm$ 2 h and $\pm$ 4 h about the true time. In the bottom row, we plot the fraction of correctly predicted samples for each study vs. the size of the error for the DCPR model (red), CYCLOPS (blue), CHIRAL (green), and Cyclum (yellow)

In this study, we develop DCPR, a deep-learning framework for circadian phase reconstruction from untimed transcriptomic data (Fig. 1A–B). DCPR takes an expression matrix of possible cycling genes as input and employs a multi-stage representation learning framework to achieve accurate circadian phase prediction. The process begins with an initial sample alignment using PCA-based embedding, followed by joint optimization of two components: (1) gene-sample associations via deep matrix factorization (Fig. 1C), and (2) gene-gene correlations through multi-head attention (Fig. 1D). The integrated representations are then nonlinearly transformed into an enhanced feature space and projected into a 2D latent space via an autoencoder, from which the final circadian phase is inferred. We first assessed the performance of DCPR using both simulated and real transcriptomic datasets. We then compared DCPR’s performance with other existing methods, including CYCLOPS, CHIRAL, and Cyclum. Finally, we applied DCPR to three untimed transcriptomic datasets comprising 157 human brain samples, including 72 from patients with Alzheimer’s disease (AD) and 85 from healthy controls. Our analysis revealed six distinct patterns of circadian alteration in three regions of AD brain, corresponding to different stages from early to late. In summary, DCPR enables accurate circadian phase prediction and circadian pattern reconstruction from untimed transcriptomic data.

## Results

### Overview of DCPR

The DCPR framework (Fig. 1) analyzes an expression matrix of cycling genes by integrating deep learning models with a dimension reduction strategy (*see Methods*). This approach projects all samples into a 2D embedding space, enabling more accurate inference of their circadian phase. DCPR is applied to several datasets with varying sample sizes to demonstrate its ability to accurately estimate the circadian phase for each sample from untimed data, thereby facilitating the reconstruction of circadian rhythms.

### Evaluations of DCPR on synthetic data

To evaluate prediction performance, we constructed twelve synthetic datasets: nine with full circadian cycle (24 h or 48 h) and three with an incomplete cycle (14 h or 18 h), as detailed in Table 1. Each contained 1,000 genes comprising both rhythmic and non-rhythmic genes. The generated time-course data simulate the oscillatory dynamics of gene expression with different sampling intervals, experimental replicates, and various levels of noise. The simulated datasets showed low false positive rates under rigorous statistical thresholds, with only a very small number of non-cycling genes being falsely identified as rhythmic. (Supplementary Table 1).

**Table 1:** Description of the twelve synthetic datasets

Datasets	Time span	Interval	Missing time points	Replicates	No. of samples
SynDST1	24 h	2 h	0	2	24
SynDST2	24 h	4 h	0	3	18
SynDST3	24 h	2 h	2	2	20
SynDST4	24 h	Non-uniform	0	1	24
SynDST5	48 h	1 h	0	1	48
SynDST6	48 h	2 h	0	2	48
SynDST7	48 h	4 h	0	3	36
SynDST8	48 h	2 h	3	2	42
SynDST9	48 h	Non-uniform	0	1	48
SynDST10	14 h	1 h	0	1	14
SynDST11	18 h	1 h	0	1	18
SynDST12	18 h	2 h	0	2	18

We applied DCPR to the above synthetic datasets and computed all the evaluation metrics, including the Area under the curve (AUC) of the error cumulative distribution function (CDF), Median Absolute Error (MedAE), etc. (Supplementary Table 2). On the first nine datasets with full cycle, DCPR achieved an average AUC of 0.969 across all datasets, outperforming CHIRAL (0.951), Cyclum (0.932), and CYCLPOS (0.687). Although the presence of missing data may cause performance degradation, its impact on DCPR is only about 1.39%. When dealing with non-uniform datasets, DCPR and CHIRAL exhibit more robust performance and proved to be generally superior to Cyclum and CYCLOPS. On the last three datasets with incomplete cycle, DCPR outperformed three SOTA models. Although all methods exhibited performance degradation, DCPR maintained relatively high accuracy. This result confirms the challenge of learning from incomplete cycles and, importantly, establishes DCPR as a reliable choice for modeling data from study with a sampling time span shorter than 24 h. Moreover, Supplementary Fig. 1 demonstrates DCPR’s prediction accuracy, showing close alignment between real and predicted time points (mean MedAE = 0.562 h). Overall, comprehensive analysis of twelve simulated datasets confirmed the robustness of DCPR across varying experimental conditions (Supplementary Fig. 2).

### Circadian phase reconstruction based on homologs of genes

Following validating DCPR on simulated datasets, we further evaluated DCPR’s performance using three time-series transcriptomic datasets (GSE223761 [27], GSE56931 [28], GSE161566) derived from *Rattus norvegicus* (rat) and *Homo sapiens* (human) (Table 2). To identify seed genes for DCPR modeling, we analyzed two *Mus musculus* (mouse) datasets (GSE54651 [29], GSE25585 [30]) and one *Papio anubis* (olive baboon) dataset (GSE98965 [31]), using the non-parametric algorithm JTK_CYCLE at CircaKB online platform [32], and identified 110, 592, and 711 rhythmically expressed genes from each, respectively ($p<0.01$). Based on evolutionary conservation, these genes were then mapped to their rat and human orthologs using Homologene [33], resulting in 118, 453, and 528 high-confidence seed genes for DCPR modeling (Supplementary File 1).

**Table 2:** Six representative time-course transcriptomic datasets for performance evaluation and ablation study

Datasets	Time span	Interval	Time index	Sample size	Organism
GSE223761	24 h	4 h	2, 6, 10, 14, 18, 22	6	*Rattus norvegicus*
GSE56931	24 h	4 h	0, 4, 8, 12, 16, 20	54	*Homo sapiens*
GSE161566	24 h	2 h	24, 26, …, 44, 46	12	*Homo sapiens*
GSE11923	48 h	1 h	18, 19, …, 64, 65	48	*Mus musculus*
GSE50438	48 h	4 h	0, 4, 8, …, 40, 44	72	*Arabidopsis thaliana*
GSE259431	48 h	2 h	45, 47, …, 89, 91	96	*Triticum aestivum*

**Fig. 3:**
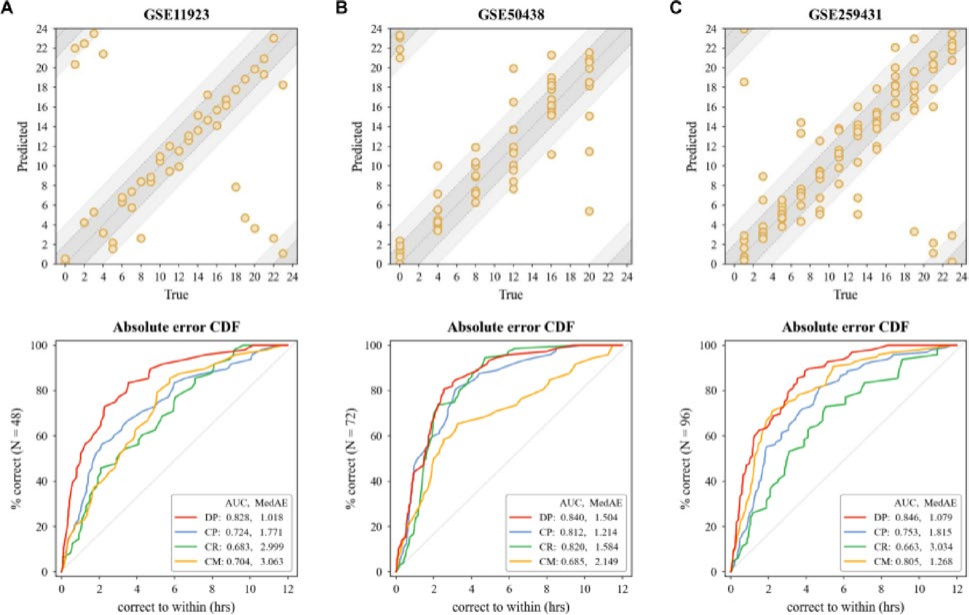
Accuracy of DCPR without prior knowledge applied to three real datasets **A** GSE11923, **B** GSE50438, and **C** GSE259431. Each column corresponds to the sample plot and AUC curve for one dataset. In the top row, the predicted time of day vs. true time of day for each sample is shown. Dark and light gray bands indicate an error range of $\pm$ 2 h and $\pm$ 4 h about the true time. In the bottom row, we plot the fraction of correctly predicted samples for each study vs. the size of the error for the DCPR model (red), CYCLOPS (blue), CHIRAL (green), and Cyclum (yellow)

As shown in Fig. 2, DCPR achieved AUC values of 0.803–0.897 across the above three datasets, significantly outperforming other state-of-the-art (SOTA) methods. The median absolute difference (MedAE) between predicted and actual experimental time points ranged from 0.803 to 1.179 h. Among all the SOTA models, CHIRAL (~ 0.741) and Cyclum (~ 0.736) are behind to our DCPR model. While CYCLOPS only showed comparable performance on the GSE223761 dataset, its accuracy declined substantially on the other two datasets (Supplementary Table 4, Fig. 3).

### DCPR performance without seed gene selection

To rigorously assess the generalizability of DCPR, we evaluated its performance using all genes as input without relying on homology-based seed gene selection. We analyzed the proposed model to three time-series transcriptomic datasets from mouse (GSE11923 [34]), Arabidopsis (GSE50438 [35]), and wheat (GSE259431) (Table 2). These datasets were specifically chosen due to the lack of corresponding time-series data from homologous species, presenting a challenging but biologically realistic test scenario of DCPR’s robustness.

As shown in Fig. 3, DCPR still achieved high temporal accuracy, with most of the samples predicted within the error range of $\pm$ 4 h of their actual time points. Remarkably, despite the inclusion of a substantial portion of non-circadian genes, DCPR maintained strong performance with AUC values ranging from 0.828 to 0.846. This stability contrasts to the three SOTA models, which presented reduced performance under the same conditions (Supplementary Fig. 4). These results highlighted DCPR's distinctive robustness and its ability to accurately infer circadian phase across diverse genomic contexts, even in the absence of prior knowledge from homologous species.

### Circadian pattern identification of clock genes using real data

Building on our evaluation of DCPR’s overall phase reconstruction accuracy, we next assessed its effectiveness in recovering the periodic patterns of known circadian genes (see *Methods*). As an example, we analyzed the GSE11923 dataset to determine whether DCPR’s prediction aligns with the prior knowledge from the Circadian Gene Database (CGDB) [36]. Previous research has shown that the liver-specific circadian clock plays a critical role in regulating sleep disorders and metabolic diseases, with genome-wide profiling revealing liver-specific circadian rhythms [32]. The CGDB documents 4,334 cycling genes in mouse liver that have been experimentally validated and supported by multiple studies [37–41].

**Fig. 4:**
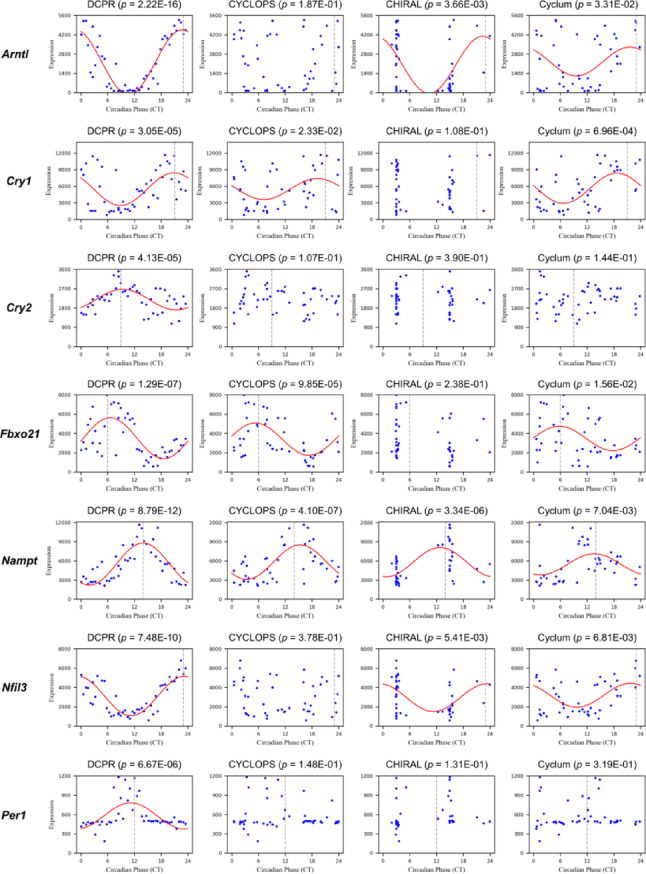
Reconstruction of circadian patterns for seven clock genes. Each row corresponds to the reconstructed circadian pattern of one gene predicted by DCPR. CYCLOPE, CHIRAL, and Cyclum. The dash line denotes the true peak (acrophase)

To evaluate phase inference accuracy, we reordered samples using phases predicted by DCPR and three other methods. This analysis identified at least 2,898 genes exhibiting rhythmic expression, corresponding to 3,996 prior-knowledge entries from CGDB, which document experimentally validated circadian parameters (*e.g*., peak and trough). Among these, 730 records aligned with DCPR’s predictions within ± 1 h, surpassing the performance of CYCLOPS (545), Cyclum (549), and CHIRAL (579) (Supplementary Table 4). Moreover, we analyzed 20 core clock genes (CCGs) and found that 19 exhibited significant periodicities under the phase predicted by DCPR, compared to 7 for CYCLOPS, 11 for Cyclum, and 11 for CHIRAL. For each CCG, we selected the most reliable prior knowledge reference based on the minimal MAE across all four methods. As summarized in Supplementary Table 5–6, DCPR’s prediction for the peak and trough times of 14 CCGs most closely matched prior knowledge, significantly outperforming CYCLOPS (1), Cyclum (3), and CHIRAL (2). Figure 4 illustrates the reconstructed circadian patterns of 7 core clock genes under DCPR’s predictions, all showing peak/trough timing errors of less than 1 h, further supporting the model’s accuracy. More Details are shown in Supplementary File 2.

### Circadian variation of gene expression identified from untimed transcriptome datasets

In this study, we applied DCPR to investigate the circadian variation of gene expression associated with Alzheimer’s Disease (AD). Specifically, we analyzed 3 untimed transcriptomic datasets comprising 85 healthy donors and 72 AD patients, spanning three brain regions: Entorhinal Cortex (EC), Hippocampus (HIP), and Frontal Cortex (FC) (Table 3). It is well established that the EC exhibits the earliest histological alterations in AD, with its functional impairments progressively disrupting hippocampus connectivity and contributing to memory deficits [42–45]. In contrast, frontal cortex degeneration is associated with late-stage behavioral cognitive decline [46]. Therefore, we investigated the above datasets in the order of EC $\to$ HIP $\to$ FC to reflect the typical progression of AD pathology. Samples from each condition were reordered based on the circadian phases inferred by DCPR and fitted to a cosinor regression model. To assess differences in rhythmicity, we defined six distinct circadian variation patterns (see *Methods*). Using the proposed framework, we analyzed 18 core clock genes, including *CLOCK*, *ARNTL*, *CRY1*, *CRY2*, and others (Supplementary File 3).

**Table 3:** AD-related untimed transcriptomic datasets collected from three regions of human brain

Brain region	Total samples	Normal	AD	GEO ID	Data profiles
Entorhinal Cortex (EC)	23	13	10	GSE5281	GSE5281_EC
Hippocampus (HIP)	63	32	31	GSE29378	GSE29378_HIP
Frontal Cortex (FC)	71	40	31	GSE15222	GSE15222_FC

**Fig. 5:**
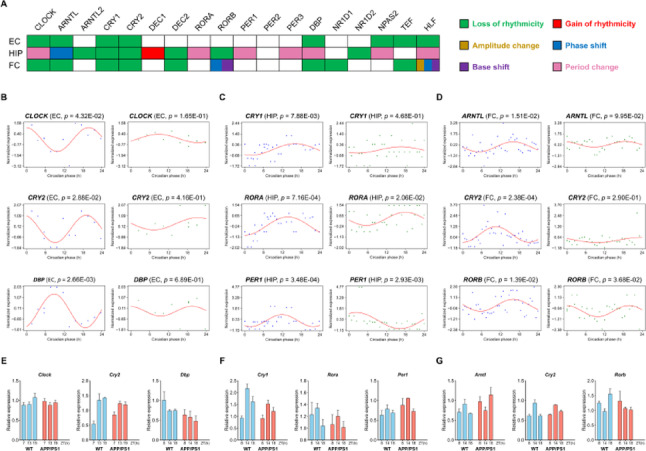
DCPR analysis of circadian alterations in early and late on set of AD. **A** the distribution of circadian changes of clock genes in three brain regions (EC, HIP, and FC) detected by DCPR. Based on DCPR-predicted phase, the circadian rhythms of nine representative clock genes from three regions, **B** EC, **C** HIP, and **D** FC, are reconstructed. The dots with blue and green color represent control and AD samples, respectively. **E** Real-time qPCR analysis of *Clock* and *Cry2* were achieved by Carrero and colleagues, et al., with the cortex of 6-month APP/PS1 mouse model. The RNA expression of *Dbp* were observed by Yang, et al., through the cortex of 2-month APP/PS1 model. **F**-**G** Two groups of clock gene were observed by Yang, et al., through the hippocampus and cortex of 10-month APP/PS1 model

As presented in Fig. 5A, most genes predicted by DCPR showed either a loss of rhythmicity or period change across different brain regions (Supplementary Table 7). Two genes (*RORB* and *HLF)* in the FC displayed multiple types of changes in circadian patterns. In particular, Fig. 5B-D highlights nine core clock genes with significant rhythmic alterations in three brain regions associated with AD progression. Specifically, *CLOCK*, *CRY2*, and *DBP* exhibit a pronounced loss of rhythms in the EC of the AD group (Fig. 5B). In the Hippocampus of AD, *CRY1* shows a notable loss of circadian rhythm, while both *RORA* and *PER1* display changes in circadian period (Fig. 5C). Notably, In the FC, *ARNTL* and *CRY2* loss rhythmicity in AD samples, whereas *RORB* exhibits both a phase shift and a base shift (Fig. 5D).

To validate these findings, we collected and quantified data from real-time qPCR experiments generated by other researchers using cortical and hippocampal tissues from APP/PS1 transgenic mice [47, 48]. As shown in Fig. 5E-G, the diurnal expression patterns of these genes observed in wide-type (WT) mice and AD mice closely align with our predicted trends for two groups of human brain samples. Specifically, the circadian rhythms of six clock genes (*e.g*., *Clock*, *Cry1*, *Cry2*, etc.,) are markedly diminished in the AD group. Three genes (*Per1, Rora, and Rorb*) still retained rhythm in AD mice, but their rhythmic patterns are altered. Overall, these findings demonstrated that DCPR could effectively uncover temporal dynamics of gene expression in existing datasets, offering a valuable approach for advancing circadian biology toward clinical applications.

### Contribution of DCPR’s architecture to its performance

**Fig. 6:**
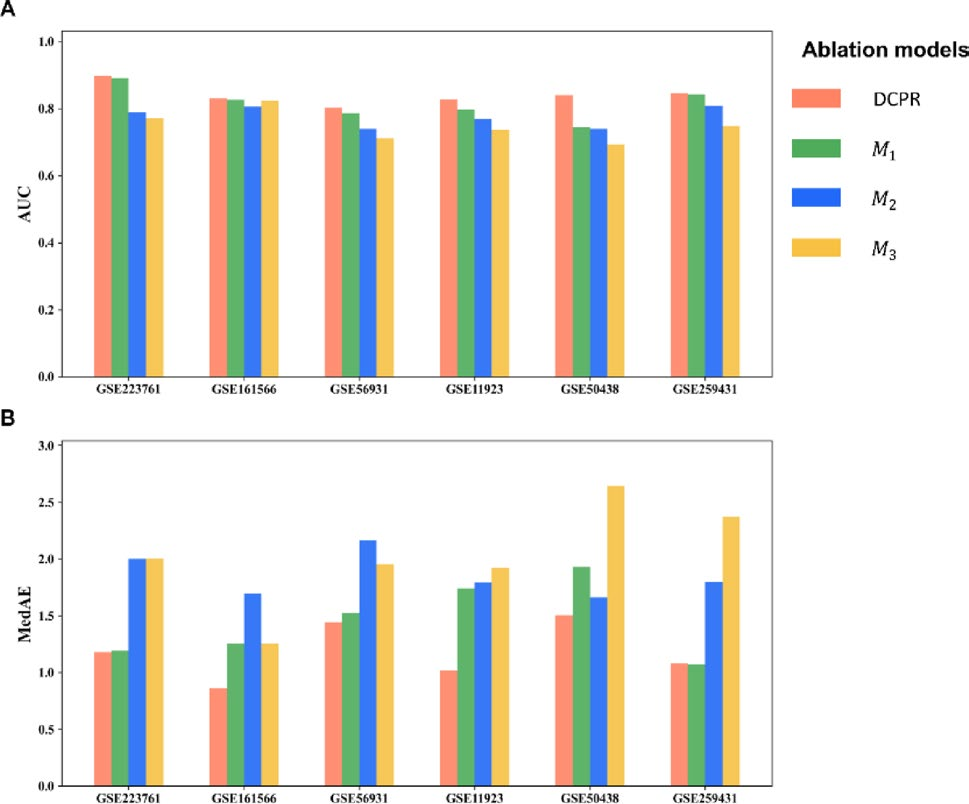
Performance evaluation of DCPR and three variants (${M}_{1}$, ${M}_{2}$, and ${M}_{3}$) across six GEO datasets. Two metrics **A** AUC and **B** MedAE are evaluated. ${M}_{1}$: DCPR model without the data augmentation module; ${M}_{2}$: DCPR model without the multi-stage representation module; ${M}_{3}$: DCPR model without both modules

To understand how DCPR’s architectural design contributes to its strong performance, we implemented an ablation study using six GEO datasets referenced in Figs. 2–3 (see *Methods*). As shown in Fig. 6, both the data augmentation module and the multi-stage representation module positively impact prediction accuracy. Particularly, the multi-stage representation learning framework had a greater influence on performance than data augmentation. According to Supplementary Table 8, removing the data augmentation module only results in a 3.11% decrease in AUCs, while replacing DCPR with a standard AE model results in a more substantial 7.74% reduction in accuracy. In addition, Supplementary Table 9 shows that applying the DCPR-derived preprocessing (DP_pre) to SOTA models improved their performance, indicating the general utility of the PCA-based sample pre-sorting and data augmentation modules in enhancing circadian phase inference. These findings highlight the importance of DCPR’s innovative architecture in achieving its high performance.

## Discussion

We present DCPR, an unsupervised deep learning framework designed to infer the circadian phase from untimed transcriptomic data accurately. Different from supervised learning approaches [49–51], DCPR leverages the intrinsic data structure without relying on external time labels. Through a comprehensive evaluation on both simulated and real datasets, DCPR demonstrates excellent performance and broad generalizability. Compared to three SOTA methods, DCPR provides more accurate phase estimation. Moreover, experimental validation of DCPR-guided predictions using mouse liver data and AD brain data supports the reliability of DCPR in reconstructing circadian gene expression patterns from untimed samples.

Comprehensive analysis of synthetic datasets indicates that DCPR remains highly robust under varying experimental conditions (*e.g*., time span, sampling interval, and noise levels), outperforming three SOTA models (Supplementary Fig. 5). Given that real transcriptomic data often contains substantial imperfections due to experimental and technical variability, these findings highlight DCPR's high potential for reliable phase prediction in practical applications.

To our knowledge, this study is the first comprehensive evaluation of the predictive performance of multiple representative methods across a wide range of real time-course transcriptomic datasets (Supplementary Fig. 6). While Anafi et al. reported that CYCLOPS revealed circadian patterns of core clock genes across three datasets, quantitative accuracy metrics were not provided. Similarly, CHIRAL has only been tested on a time-course dataset (GSE108539), and its generalizability remains largely unexplored. Additional testing on two datasets from these algorithms further showed the reliability of DCPR (Supplementary Table 10). Therefore, our study provides novel insight that enables a more reliable algorithm selection for circadian phase inference.

A key advantage of DCPR illuminated by this evaluation is its robustness to sample size and sampling interval, enabling flexible application across diverse scenarios. It consistently delivered excellent performance across datasets of varying scales, in contrast to CYCLOPS and CHIRAL, which have notable sample size requirements. As mentioned by Anafi et al., CYCLOPS typically requires more than 250 samples to reliably order circadian phases [23]. DCPR is not significantly affected by data quantity, which highlights its practical utility for phase reconstruction with limited sample size.

Most importantly, we tested the models under a fundamentally challenged condition in which the circadian system is disrupted. Initial assessment on the Bmal1 knockout dataset (GSE196430) showed that while all methods performed well on wild-type (WT) samples, their accuracy decreased markedly in knockout (KO) samples (Supplementary Table 11). Despite this, DCPR maintained superior performance, demonstrating initial promise. To extend this finding, we evaluated the models on a larger, multifactorial dataset (GSE135898), in which samples are categorized into eight groups based on genotype (Cry1/2 WT/KO and Bmal1 WT/KO) and feeding regimen (AL or NRF) (Supplementary Table 12). Consistently, DCPR achieved the best AUC in seven of the eight groups under both WT and KO conditions. In contrast, while CHIRAL's performance was close to DCPR's under WT conditions, it declined markedly in clock-disrupted mice. This result confirms that CHIRAL’s design fundamentally relies on the core clock genes of organisms with an intact, healthy circadian system.

Seed gene selection plays a critical role in the DCPR framework by enriching rhythmic genes to ensure accurate phase prediction. Although DCPR tolerate input data containing noise, missing timepoints, uneven sampling, and non-oscillatory signals, incorporating seed genes significantly enhances predictive performance (Supplementary Table 3). Moreover, modeling with seed genes improves computational efficacy by accelerating the convergence of the training process (Supplementary Fig. 7).

The practical utility of DCPR to AD transcriptomic datasets highlights its potential to accurately reconstruct circadian gene expression patterns using existing untimed data. Analysis of 18 core clock genes across three brain regions revealed region-specific circadian alterations, including both single and combined changes in rhythmic patterns. Gene expression data from AD mouse brain tissues further supported the model’s predictions for nine key genes, providing insights that may inform clinical therapeutic strategies. In addition, our analysis revealed that the circadian variations of the same gene can vary significantly across different brain regions.

Despite its strengths, DCPR also has several limitations. First, while homology-based conservation is useful for selecting seed genes, it does not guarantee that all selected genes are cycling, which may introduce noise and affect model performance. Second, the relatively complex architecture of DCPR may increase computational and training costs. Lastly, the DCPR's effectiveness should be validated across broader application scenarios to fully establish its generalizability.

## Conclusions

In this study, we proposed DCPR, a novel deep learning framework for circadian phase estimation. We show this model using extensive time-course simulated and real data and that it gives remarkably impressive results when applied to samples of different scales across multiple species. Then, we confirm its effectiveness in recovering the circadian rhythms of clock genes in normal mouse liver. Finally, we demonstrate DCPR’s potential of further identifying circadian variation of gene expression in AD progression from untimed transcriptome datasets. DCPR provides a powerful tool for temporal reconstruction, facilitating investigations of circadian biology in disease contexts.

## Methods

### Simulations on synthetic datasets

To evaluate DCPR’s performance in recovering circadian phases from untimed samples, we conducted a simulation study using twelve synthetic datasets with 1000 genes. These datasets are used to assess the impact of time span (full circadian cycle, or incomplete cycle), sampling interval, the presence of missing observations, number of replicates, and noise intensity on model performance (Table 1). Given a time step collection $T=[{t}_{1},\cdots ,{t}_{i},\cdots ,{t}_{L}]$, we generate 200 genes that are likely to be rhythmic as:

1 $$f=A*\mathrm{cos}\left(\frac{2\pi }{24}*[T+\varphi ]\right)+(\frac{A}{\sigma }*S)$$

where $S=[{s}_{1},\cdots ,{s}_{i},\cdots ,{s}_{L}]$ denotes a Gaussian noise series with $s_{i} \sim N(0, 1)$. In addition, $\sigma$ represents Signal–Noise-Ratio (SNR) and follows a uniform distribution $\sigma \sim U\left( {2,5} \right)$. For each above gene, the circadian amplitude $A$ and phase shift $\varphi$ were randomly set as $A\sim U(0.5,1)$ and $\varphi \sim U(0,24)$ respectively. For the non-cycling component, we generated 800 genes by simulating their expression profiles as Gaussian noise ($f=A*S$), where $A$ is similarly drawn from $A\sim U(0.5,1)$.

### Public datasets collection and preprocessing

To further evaluate the performance of the DCPR model on phase reconstruction, we selected ten time-series transcriptomic datasets from Gene Expression Omnibus (GEO), covering multiple species (GSE223761, GSE56931, GSE161566, GSE11923, GSE50438, GSE259431, GSE71620, GSE108539, GSE196430, and GSE135898). Particularly, the first three datasets are used to assess the effect of homology-based seed gene selection on the improvement of model outcome, while the next three are employed to evaluate DCPR’s performance without prior knowledge (Table 2). Moreover, three untimed AD datasets (GSE5281, GSE29378, and GSE15222) were collected to explore the potential of DCPR in phase reconstruction (Table 3). All the above datasets were downloaded from GEO and underwent z—normalization.

### DCPR modeling

As an unsupervised model designed for global structure discovery, DCPR processes the entire dataset of unlabeled samples to reconstruct a unified circadian timeline. Its core output is a precise phase assignment on a circular embedding for each sample. This phase order is determined through a global optimization process that relies on the interrelationships among all samples. DCPR modeling consists of the following steps:

1. Seed gene selection.

Based on Fourier series and Principal component analysis (PCA) [52, 53], we propose a *mathematical inference for phase reconstruction*: 1) Any periodic signal meeting the Dirichlet conditions is decomposable into a trigonometric series of sine and cosine components; 2) PCA applied to isochronous periodic variables reveals that the first two components (${PC}_{1}$, and ${PC}_{2}$) constitute an orthogonal basis of cosine and sine functions, with data projections forming a circular pattern in this embedding space.

To infer circadian phase, our DCPR model projects untimed high-dimensional samples into a 2D embedding space and predicts their optimal polar angle (0–2 $\pi$) on the unit circle to determine circular position. This projection is preferentially based on a set of seed genes, when a time-course dataset from a homologous species and matched tissue is available; otherwise, it uses all genes. For the former case, the seed genes are derived from cycling genes that are identified from the reference data under a rigorous statistical threshold (*e.g*., $p<0.01$ or $q<0.05$) and subsequently filtered by homology matching.

(2) Sample pre-sorting based on PCA analysis.

Since seed genes in matrix $X$ are more likely to cycle, all samples are embedded into a unit circle via PCA analysis. The circular position of $i$-th sample is given by:

2 $${\theta }_{i}=\mathrm{arctan}({PC}_{2}/{PC}_{1})$$

where ${PC}_{1}$ and ${PC}_{2}$ are the top 2 principal components. Based on ${\theta }_{i}$, we implement sample pre-sorting and obtain matrix $Z$.

(3) Data augmentation.

To guarantee the periodic patterns of genes input into the DCPR model, we use the expression profile $Z\left(i,\right)$ of gene $i$ to fit the cosinor model and obtain the predicted series $Y\left(i,\right)$:

3 $$Y\left(i,\right)=\mathrm{cos}\left(Z\left(i,\right);\theta \right)+{\varepsilon }_{i}$$

where $\theta$ and ${\varepsilon }_{i}$ are the model parameters and fitting residuals, respectively. The approximate matrix $Y$ will be used for DCPR modeling. In the absence of prior knowledge of homologous species, we suggest users to input all genes and select the first 1,000 genes with the smallest *p*-value to construct matrix $Y$ to ensure computational efficiency.

(4) DCPR modeling.

Figure 1B presents the architecture of DCPR model. Based on the approximate matrix $Y$, we first calculated the gene-sample association matrix $S$ and gene–gene correlation matrix $G$. And then, the Dual-context matrix $E$ can be obtained through $E=[{S}^{T};G]$. A fully connected network with five layers decomposes the input $E$ and outputs a reconstructed matrix $F$. Finally, the matrix $F$ is used as the input of an autoencoder model and improves the circular representation in 2D latent space.

### Identifying gene-sample association using deep matrix factorization

As shown in Fig. 1C, the matrix $Y$ and its transpose ${Y}^{T}$ are input into two five-layer neural networks respectively, obtaining the feature matrices ${HG}_{2*M}$ and ${HS}_{2*N}$ for all genes and samples in a two-dimensional space. The gene-sample association matrix $S$ can be expressed as: $S={HG}^{T}*HS$. Since the neural network architectures for solving $HG$ and $HS$ are similar, we only use the solution process of $HG$ as an example for explanation. In Fig. 1C, the number of neurons in the input layer, hidden layers, and output layer of the first neural network are: $N$, $2$, $2$, $1$, and $2$. The weight matrix of the first hidden layer ${h}_{1}$ is ${W}_{1}^{(1)}$, and the bias matrix is ${b}_{1}^{(1)}$. After the input matrix is mapped to a two-dimensional space, the second hidden layer ${h}_{2}$ is obtained through cos and sin functions. The hidden layer ${h}_{3}$ has only one neuron, which outputs the polar angles of all genes on the unit circle. The output layer outputs the coordinates of all genes on the unit circle. Therefore, the calculation steps of the feature matrix $HG$ are as follows:

4 $$({h}_{11};{h}_{12})={W}_{1}^{(1)}{Y}^{T}+{b}_{1}^{(1)}$$

5 $$ \begin{gathered} \left( {h_{21} ;h_{22} } \right) \hfill \\ = \left( {\frac{{\cos \left( {h_{11} } \right)}}{{\sqrt {\left( {\cos \left( {h_{11} } \right)} \right)^{2} + \left( {\sin \left( {h_{12} } \right)} \right)^{2} } }};} \right. \hfill \\ \left. {\quad \quad \frac{{\sin \left( {h_{12} } \right)}}{{\sqrt {\left( {\cos \left( {h_{11} } \right)} \right)^{2} + \left( {\sin \left( {h_{12} } \right)} \right)^{2} } }})} \right) \hfill \\ \end{gathered} $$

6 $${h}_{3}=\mathrm{arctan}({h}_{22}/{h}_{21})$$

7 $$HG=(\mathrm{cos}\left({h}_{3}\right);\mathrm{sin}({h}_{3}))$$

### Identifying gene–gene correlation using multi-head attention

First, the input matrix ${Y}_{M\times N}$ is multiplied by three different matrices ${W}^{Q}$, ${W}^{K}$, and ${W}^{V}$, yielding the query matrix $Q$, key matrix $K$, and value matrix $V$. Assuming the linear embedding dimension at this stage is ${d}_{0}$, a single-head self-attention can be defined as follows:

8 $$Attention\left(Q,K,V\right)=softmax\left(\frac{Q{K}^{T}}{\sqrt{{d}_{k}}}\right)V$$

Here,${W}^{Q}$,${W}^{K}$, ${W}^{V}\in {R}^{N\times {d}_{k}}$, where ${d}_{k}$ is the dimension of the low-dimensional subspace, and${W}^{O}\in {R}^{M\times h{d}_{k}}$. The multi-head attention mechanism constructs multiple subspaces to capture diverse information from the input matrix. The $i$-th head can be defined by Eq. (9–10):

9 $${head}_{i}=Attention(Q{W}_{i}^{Q},K{W}_{i}^{K},V{W}_{i}^{V})$$

10 $$ \begin{gathered} {\mathrm{MultiHead}}\left( {Q,K,V} \right) \hfill \\ \quad = {\mathrm{Concat}}\left( {{\mathrm{head}}_{1} , \cdots ,{\mathrm{head}}_{h} } \right)W^{O} \hfill \\ \end{gathered} $$

where ${W}_{i}^{Q},{W}_{i}^{K},{W}_{i}^{V}\in {R}^{{d}_{0}\times {d}_{k}}$,${W}^{O}\in {R}^{M\times h{d}_{k}}$, and ${d}_{k}$ represents the dimensionality of each head. As shown in Fig. 1D, the total number of heads $h$ is set to 2. The gene–gene correlation matrix $G$ is expressed as:

11 $$G=softmax(MultiHead\left(Q,K,V\right))$$

### Generating the deep-enhanced representation matrix

As shown in Fig. 1B, integrating the association matrix $S$ and $G$, we obtain $E$, which encodes the intrinsic relationship between samples and features in the data. A fully connected deep neural network with three hidden layers (${H}_{1}$, ${H}_{2}$, ${H}_{3}$) then accepts $E$ as input and output the enhanced representation matrix $F$. Notably, two bottleneck layers (${H}_{1}$, and ${H}_{3}$) designed in this network contribute to this feature enhancement, and their outputs can be defined as Eq. (12–13):

12 $${H}_{2}={W}_{2}^{(1)}\left(\mathrm{cos}\left({H}_{1}\left[1\right]\right);\mathrm{sin}\left({H}_{1}\left[2\right]\right)\right)+{b}_{2}^{(1)}$$

13 $${F}{\prime}={W}_{o}\left(\mathrm{cos}\left({H}_{3}[1]\right);\mathrm{sin}\left({H}_{3}[2]\right)\right)+{b}_{o}$$

where $${H}_{1}={W}_{1}^{(2)}*E+{b}_{1}^{(2)}$$, and $${H}_{3}={W}_{3}{*H}_{2}+{b}_{3}$$.

### Phase reconstruction in 2D latent space using autoencoder

After obtaining matrix $F$, the optimal positions of the samples on the unit circle of the two-dimensional embedding will be determined by two nodes in the hidden layer. The number of neurons in both the input and output layers of this AE model is $M$. The weight matrices and bias matrices are defined as ${W}_{1}^{(3)}$ and ${W}_{2}^{(2)}$, as well as ${b}_{1}^{(3)}$ and ${b}_{2}^{(2)}$. The hidden layer nodes ${l}_{1}$ and ${l}_{2}$ are defined by Eq. (14):

14 $$\left({l}_{1};{l}_{2}\right)=f({W}_{1}^{(3)}F+{b}_{1}^{(3)})$$

The output $\widehat{Y}$ of the AE model can be defined as:

15 $$\widehat{Y}=g({W}_{2}^{(2)}*\left({l}_{1};{l}_{2}\right)+{b}_{2}^{(2)})$$

After optimizing the DCPR model, the optimal positions of samples projected onto the unit circle in the two-dimensional space can be estimated based on the output values of the hidden layer nodes:

16 $${V}_{\theta }=\left(\mathrm{arctan}\left(\frac{{\partial }_{2}}{{\partial }_{1}}\right)\right)mod 2\pi $$

where $${\partial }_{1}= {l}_{1}/\sqrt{{({l}_{1})}^{2}+{({l}_{2})}^{2}}$$, $${\partial }_{2}= {l}_{2}/\sqrt{{({l}_{1})}^{2}+{({l}_{2})}^{2}}$$.

(5) Model optimization and solution.

We optimize the model parameters by minimizing the following formula (17):

17 $$\mathrm{min}({\Vert Y-\widehat{Y}\Vert }^{2}+\sum_{i=1}^{N}\frac{1}{1+{e}^{-{P}_{i*}{Q}_{i}}})$$

Here, ${P}_{i}=\sum_{j=1}^{N}{({e}^{-{\Vert {A}_{i}-{A}_{j}\Vert }^{2}/\delta })}^{2}$ and ${Q}_{i}=\sum_{j=1}^{N}{({e}^{-{\Vert {l}_{i}-{l}_{j}\Vert }^{2}/\delta })}^{2}$ represent the sample distance metrics in the original sample space and the low-dimensional embedding space, respectively. Clearly, the optimal solution of the DCPR model minimizes both the reconstruction error and the cross-entropy loss. The hyperparameters involved in DCPR and SOTA models are presented in Supplementary Table Table 13–14.

### Ablation study

The ablation study is designed to prove the validity of the proposed framework. In this study, we sequentially removed (1) the data augmentation module (Fig. 1A), (2) the multi-stage representation module (Fig. 1B), generating three ablated variants (${M}_{1}$, ${M}_{2}$, and ${M}_{3}$) for comparison with the complete DCPR framework (${M}_{0}$).

- ${M}_{1}$: only the first component removed
- ${M}_{2}$: only the second component removed
- ${M}_{3}$: both components removed

### Evaluation metric

For the time-coursed datasets, we mainly employ six metrics for evaluating the prediction performance of DCPR, including AUC of the absolute error CDF (cumulative distribution function) [26], MedAE (Median Absolute Error) [54], Standard Deviation of Absolute Error (SDAE), Circular Correlation Coefficient (CCC) [55], percent within 1 h, and percent within 2 h [56, 57]. The absolute error CDF of sample $i$ can be computed as $\left|{P}_{i}-{R}_{i}\right| mod 24$ (${P}_{i}$ and ${R}_{i}$ are the predicted and real time). Circular correlation coefficient is defined to evaluate concordance between two polar vectors [55]. Prior to metrics calculation, the predicted phases of all samples are aligned with the ground-truth by establishing an optimal reference starting point. This is achieved via a circular shift that minimizes the median absolute distance (MAD) [22] (Supplementary Fig. 8).    

### Definition of six types of circadian variation

Using DCPR on untimed datasets, we characterized rhythmicity alterations between control and AD groups, identifying six patterns:

- Loss of rhythmicity: One gene loss the rhythmicity in AD If ${P}_{c}\le 0.05$ && ${P}_{D}>0.05$.
- Gain of rhythmicity: One gene has no significant rhythmicity in control group but gain a rhythmicity in AD If ${P}_{c}>0.05$ && ${P}_{D}\le 0.05$.
- Period change: If (${P}_{c}\le 0.05$ && ${P}_{D}\le 0.05$) *&&*$\left|{T}_{c}-{T}_{D}\right|>2$
- Amplitude change/ Phase shift/ Base shift: assessed via diffCircadian [58] if (${P}_{c}\le 0.05$ && ${P}_{D}\le 0.05$) *&&* $\left|{T}_{c}-{T}_{D}\right|<2$

where [${T}_{c}$, ${M}_{c}$, ${A}_{c}$, ${LP}_{c}$, ${LT}_{c}$, ${P}_{c}$] and [${T}_{D}$, ${M}_{D}$, ${A}_{D}$, ${LP}_{D}$, ${LT}_{D}$, ${P}_{D}$] represent the circadian parameters (period, mesor, amplitude, peak, trough, *p*-value) of cosinor models on control and AD samples ordered by DCPR, respectively. The default period in cosinor2 (R package) [59] is set from 20 to 28 with a step of 1.

### Real-time qPCR data collection for DCPR’s validation

To validate the circadian rhythms of clock genes reconstructed through DCPR, we collected experimental data from the literature, which were obtained by real-time qPCR analysis of brain tissues from AD mouse models [47, 48]. The gene expression levels (mean and standard deviation) were directly extracted from the references using ImageJ 1.54 [60]. The collected consecutive time points should cover at least half a cycle. We mainly selected qPCR experimental data of the hippocampus and cortex of AD mouse models to validate DCPR’s prediction.

### Simulation environment

The codes of DCPR were developed and debugged by using TensorFlow 2.12.0, and Python 3.8.5 under the environment with 12th Gen Intel® Core™ i9-12900 K processor and 64 GB of RAM. All the transcriptomic datasets were analyzed in RStudio with R 3.6.0.

## Supplementary Information

Supplementary file 1 (DOCX 19 kb)

## Data Availability

All public transcriptome datasets (GSE223761, GSE56931, GSE161566, GSE11923, GSE50438, GSE259431, GSE71620, GSE108539, GSE196430, GSE135898, GSE5281, GSE29378, and GSE15222) analyzed in this study can be obtained from GEO at: https://www.ncbi.nlm.nih.gov/geo/. The identification of cycling genes from GSE54651, GSE25585, and GSE98965 was directly implemented on the CircaKB platform at: https://cdsic.njau.edu.cn/CircaKB. The prior knowledge of 27,964 experimentally identified circadian genes can be freely accessed from the CGDB website at: http://cgdb.biocuckoo.org/download.php. All the processed real and synthetic datasets can be downloaded from our Figshare repository at: https://doi.org/10.6084/m9.figshare.29125904.

## Abbreviations

DP: DCPR
CP: Cyclops
CR: Chiral
CM: Cyclum
AUC: Area under the curves (AUC) of error cumulative distribution function (CDF)
MedAE: Median absolute error
SDAE: Standard deviation (SD) of absolute error
CCC: Circular correlation coefficient
GEO: Gene expression omnibus
AD: Alzheimer's disease

## Code availability

The source code of the DCPR model is freely available on our GitHub repository https://github.com/NJAU-CDSIC/DCPR_V1.0. The code of all SOTA models is also provided. Supplementary Files 1–3 accompanying this paper can be accessed.
