# Supplementary material for: DCPR: a deep learning framework for circadian phase reconstruction
Source: BMC Bioinformatics. 2025 Dec 30;27:31. doi: 10.1186/s12859-025-06363-2 (PMC12866578; doi:10.1186/s12859-025-06363-2)
Supplement: Supplementary file 1 — (DOCX 19 kb) [file 12859_2025_6363_MOESM1_ESM.docx]

**Supplementary Information**

**DCPR: A deep learning framework for circadian phase reconstruction**

Xiao Han^1,2^, Xiaochen Cen^1,2^, Zhijin Li^3^, Xiaobo Zhou^4^, Zhiwei Ji^1,2,^ *

^1^College of Artificial Intelligence, Nanjing Agricultural University, No. 666 Binjiang Avenue, Nanjing, Jiangsu 211800, China

^2^Center for Data Science and Intelligent Computing, Nanjing Agricultural University, No. 666 Binjiang Avenue, Nanjing, Jiangsu 211800, China

^3^Department of Neurosurgery, The First Affiliated Hospital of USTC (Anhui Provincial Hospital), Division of Life Science and Medicine, University of Science and Technology of China, Hefei, Anhui 230036, China

^4^School of Biomedical Informatics, The University of Texas Health Science Center at Houston, 7000 Fannin Street, Houston, TX 77030, USA

*Corresponding author: Zhiwei Ji ([Zhiwei.Ji@njau.edu.cn](mailto:Zhiwei.Ji@njau.edu.cn))

**Supplementary Figures**

**
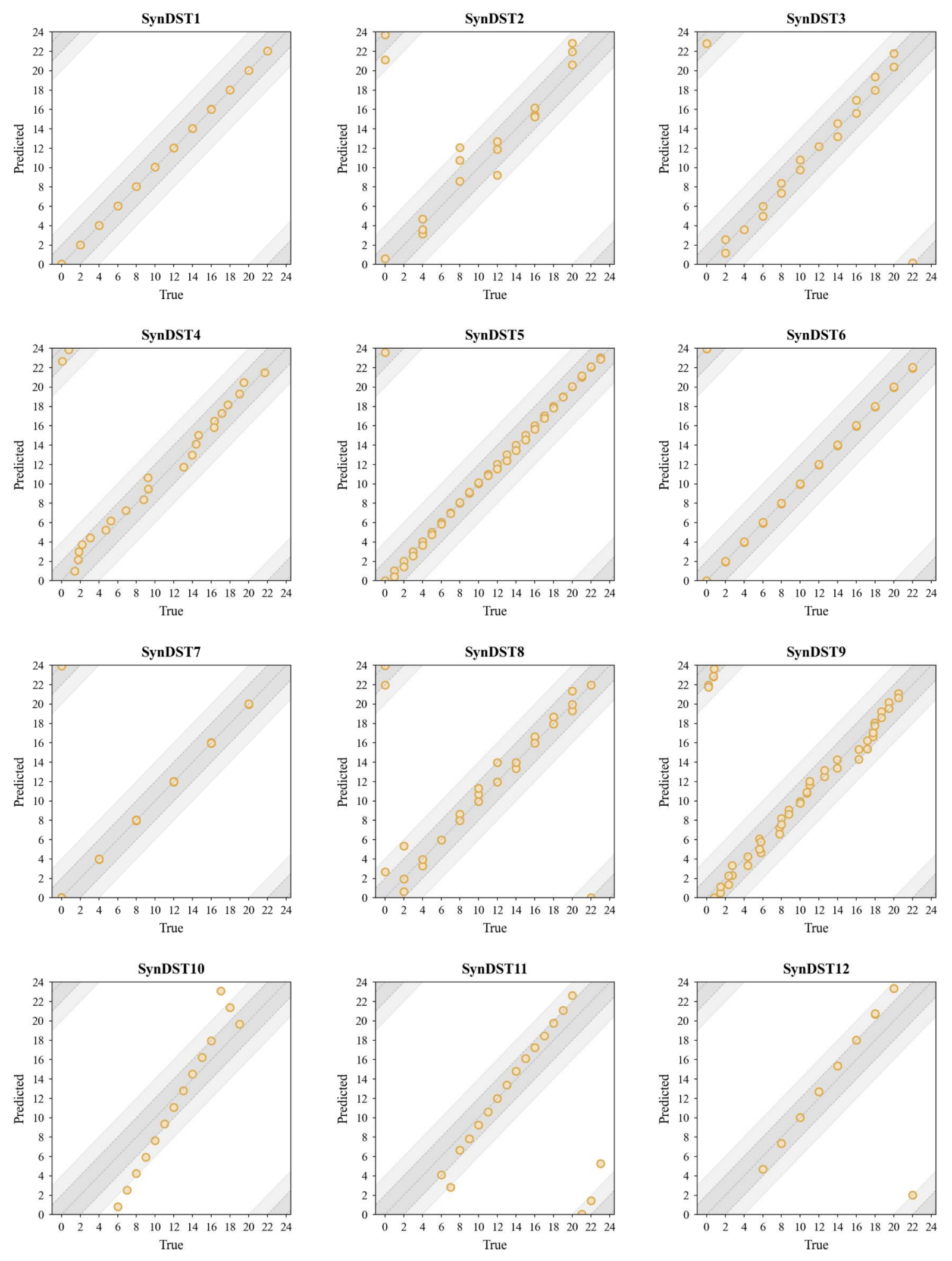
**

**Supplementary Figure 1.** Sample plots with real and DCPR-predicted time for twelve simulated datasets

**
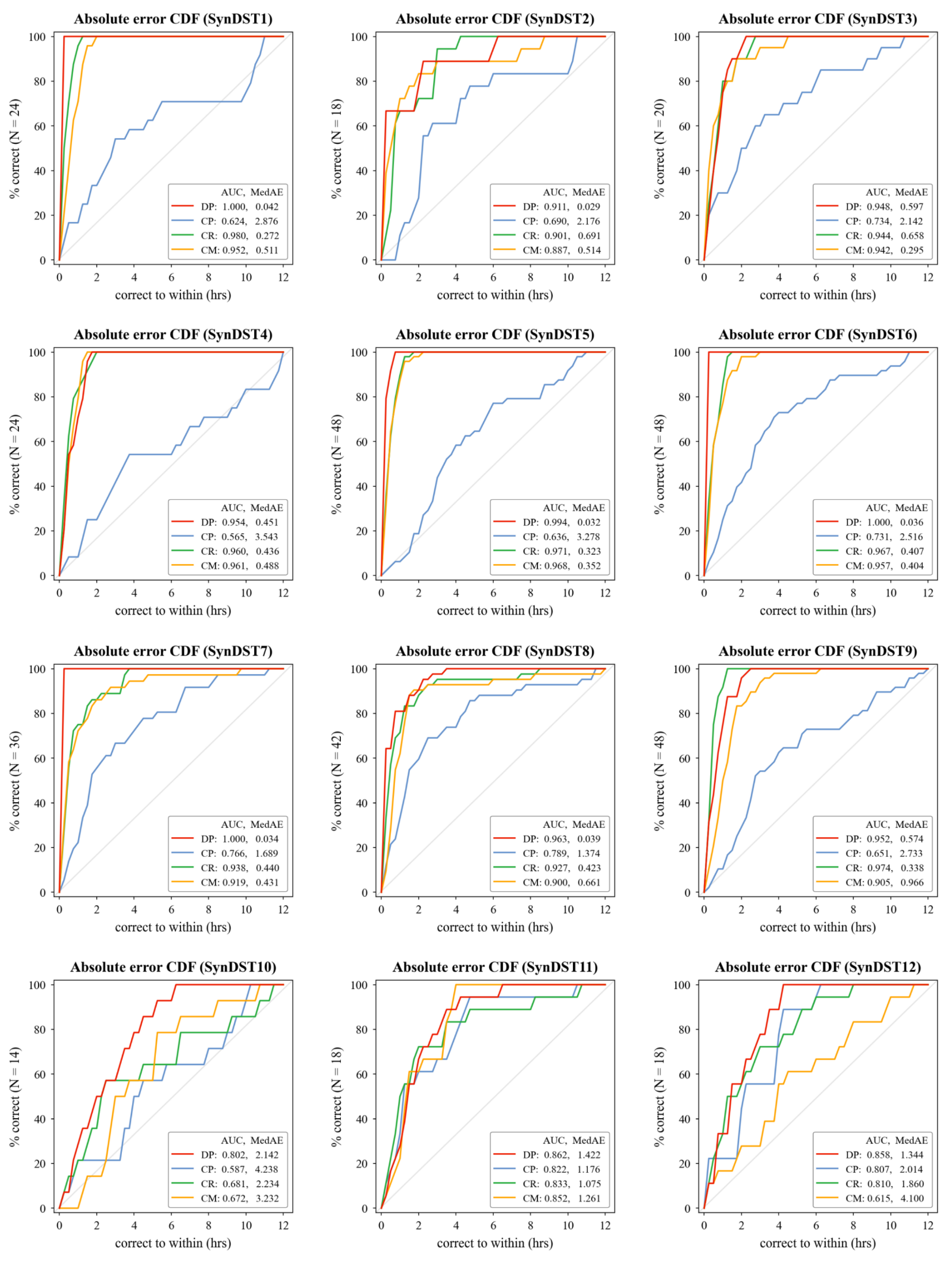
**

**Supplementary Figure 2.** Absolute error CDF curve for the phase estimates of each method on twelve simulated datasets

**
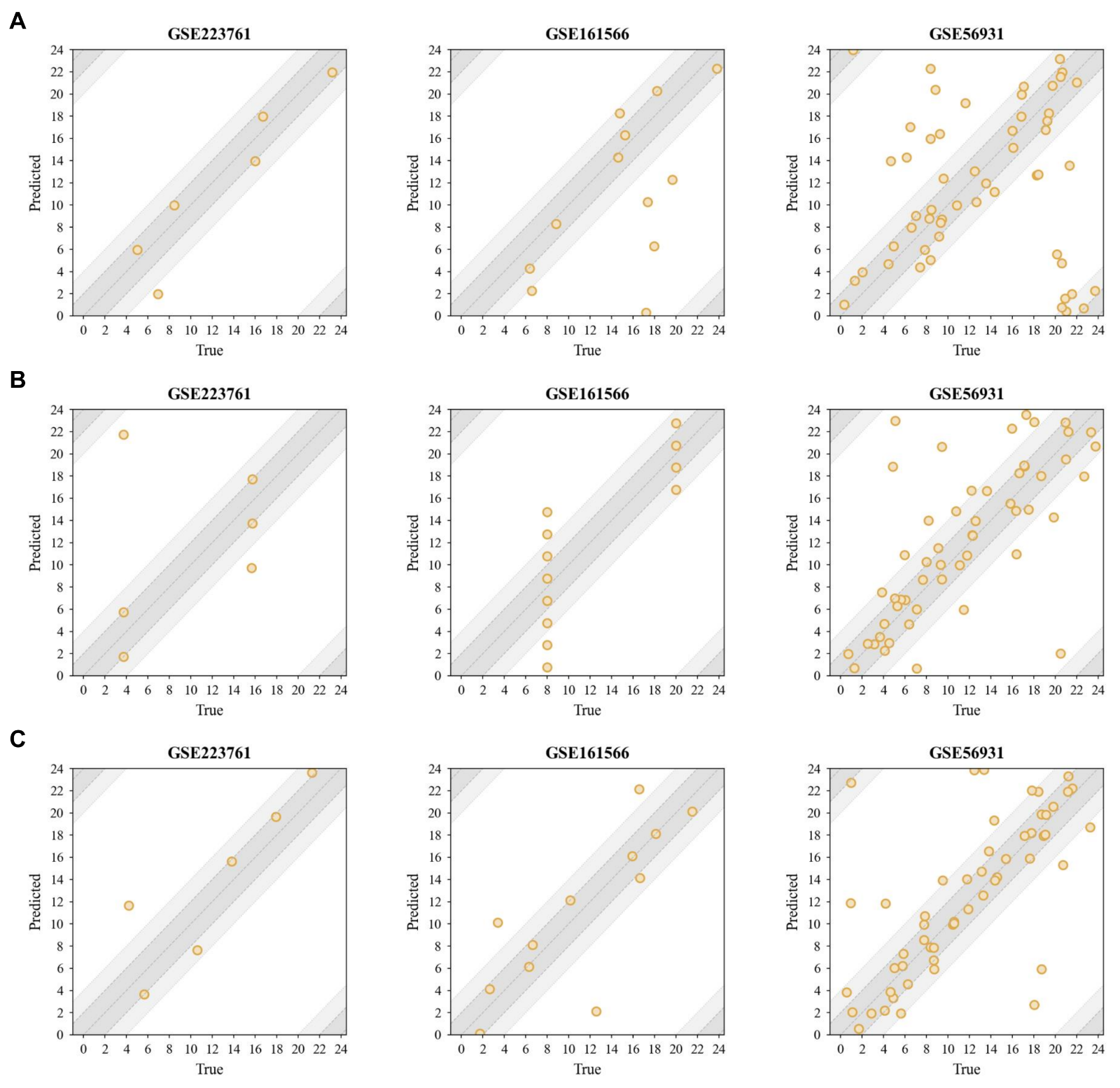
**

**Supplementary Figure 3.** Sample plots for three datasets (GSE223761, GSE161566 and GSE56931) with the real time and the time predicted by **(A)** Cyclopse, **(B)** CHIRAL, and **(C)** Cyclum

**
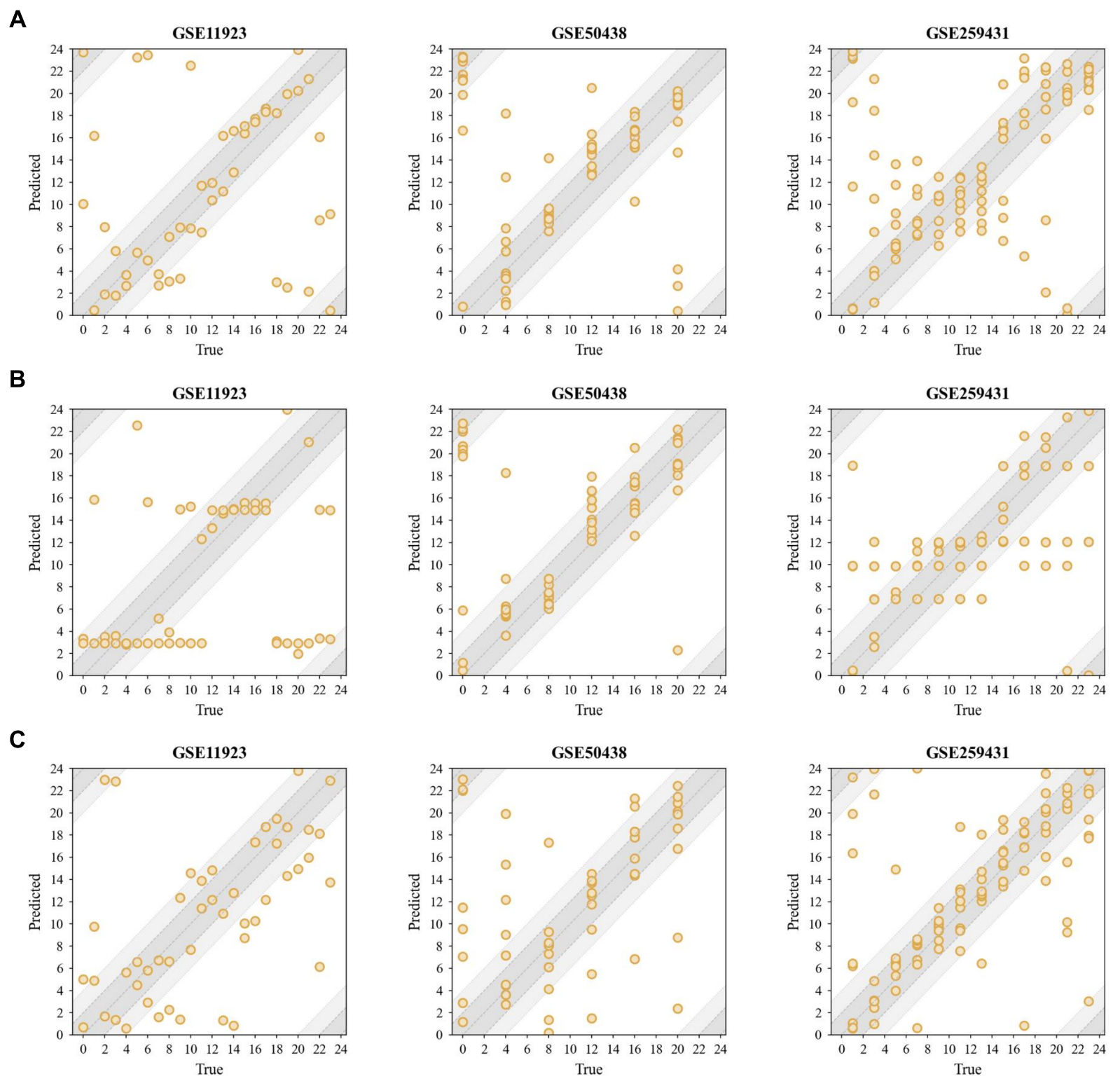
**

**Supplementary Figure 4.** Sample plots for three datasets (GSE11923, GSE50438, and GSE259431) with the real time and the time predicted by **(A)** Cyclopse, **(B)** CHIRAL, and **(C)** Cyclum

**
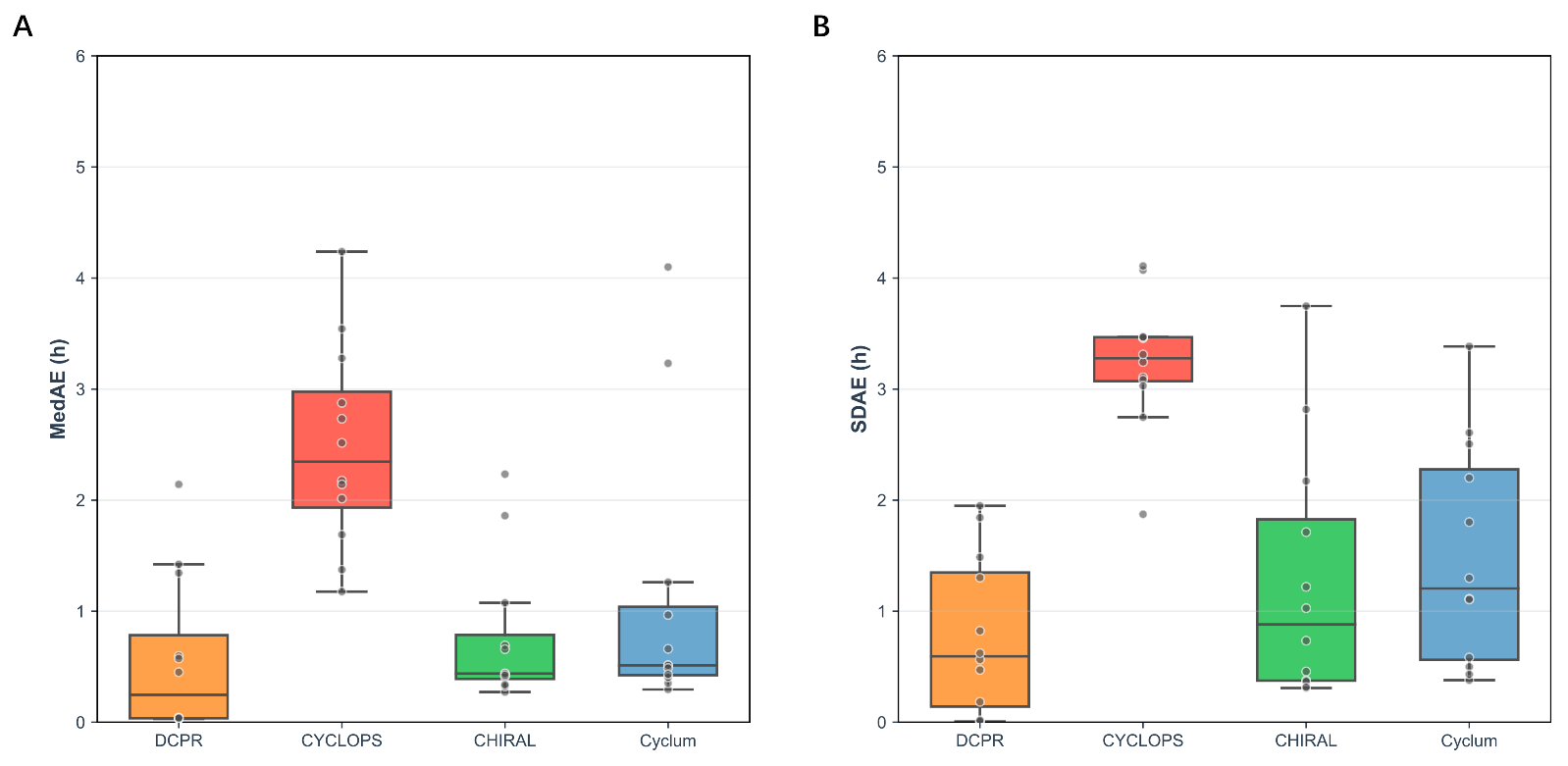
**

**Supplementary Figure 5.** Error distribution of DCPR and baselines across twelve simulated datasets: **(A)** MedAE, **(B**) SDAE.

**
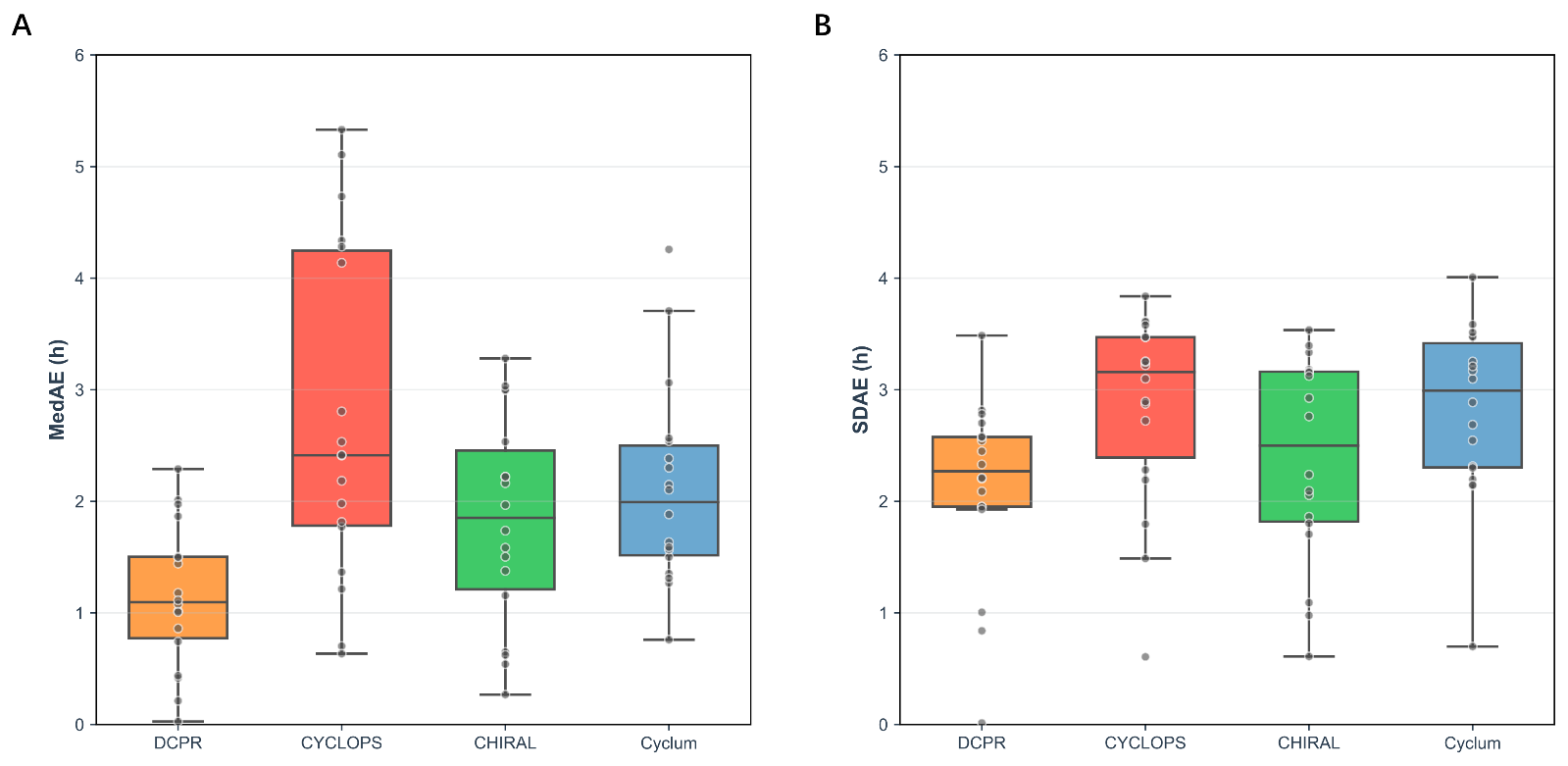
**

**Supplementary Figure 6.** Error distribution of DCPR and baselines across ten real time-course datasets from GEO: **(A)** MedAE, **(B**) SDAE.

**
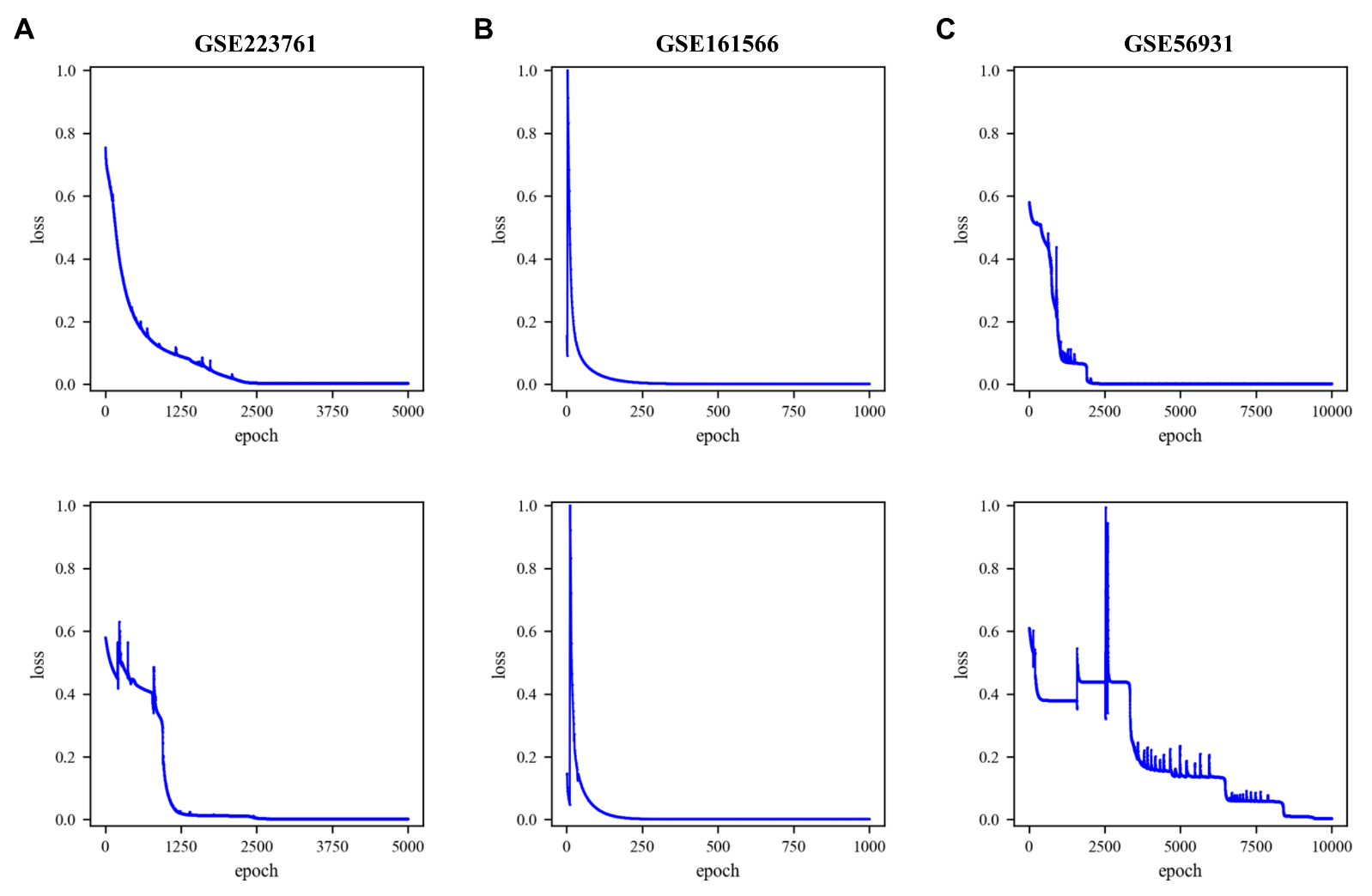
**

**Supplementary Figure 7.** The convergence of DCPR on **(A)** GSE223761, **(B)** GSE161566, and **(C)** GSE56931. The first and second rows represent the seed genes and all genes as input, respectively.

**
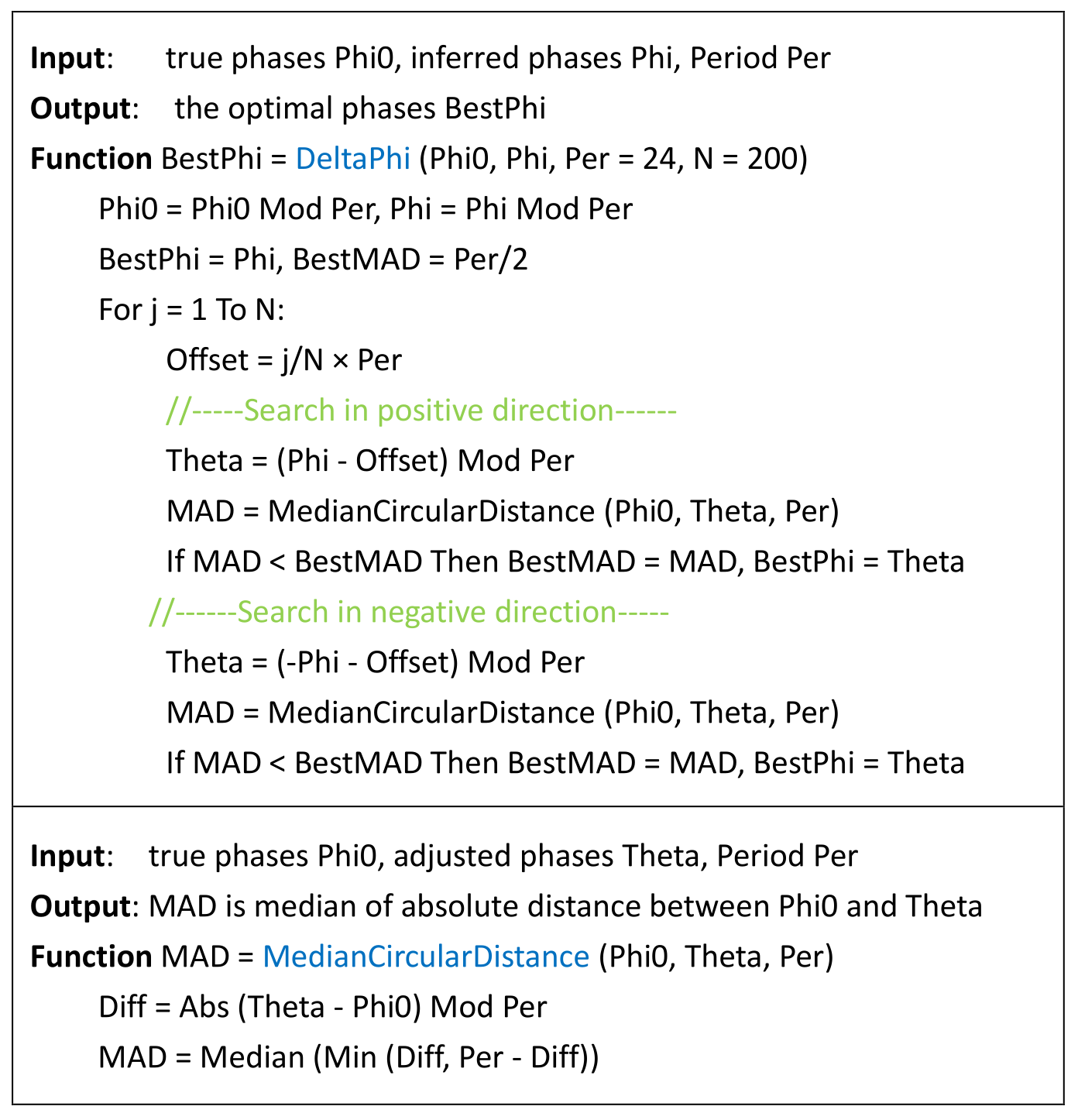
**

**Supplementary Figure 8.** The computational procedure to find an optimal reference starting point for the predicted phases of all samples.

**Supplementary Tables**

**Supplementary Table 1.** The false positive rates (FPR) for twelve simulated datasets under different significance thresholds. False positives (FP) are defined as non-cycling genes misidentified as rhythmic among the 800 non-cycling genes.

| **Datasets** | **q-value < 0.05** | | | **p-value < 0.01** | | | **q-value < 0.1** | | |
| --- | --- | --- | --- | --- | --- | --- | --- | --- | --- |
|  | **Rhythmic gene** | **FP** | **FPR** | **Rhythmic gene** | **FP** | **FPR** | **Rhythmic gene** | **FP** | **FPR** |
| **SynDST1** | 201 | 5 | 0.0063 | 201 | 5 | 0.0063 | 214 | 16 | 0.0200 |
| **SynDST2** | 4 | 0 | 0 | 86 | 10 | 0.0125 | 65 | 8 | 0.0100 |
| **SynDST3** | 197 | 8 | 0.0100 | 197 | 8 | 0.0100 | 212 | 17 | 0.0213 |
| **SynDST4** | 215 | 15 | 0.0188 | 214 | 14 | 0.0175 | 221 | 21 | 0.0263 |
| **SynDST5** | 208 | 8 | 0.0100 | 208 | 8 | 0.0100 | 217 | 17 | 0.0213 |
| **SynDST6** | 202 | 8 | 0.0100 | 202 | 8 | 0.0100 | 207 | 11 | 0.0138 |
| **SynDST7** | 9 | 0 | 0 | 98 | 12 | 0.0150 | 89 | 12 | 0.0150 |
| **SynDST8** | 190 | 7 | 0.0088 | 190 | 7 | 0.0088 | 207 | 15 | 0.0188 |
| **SynDST9** | 203 | 3 | 0.0038 | 203 | 3 | 0.0038 | 210 | 10 | 0.0125 |
| **SynDST10** | 200 | 5 | 0.0063 | 200 | 5 | 0.0063 | 214 | 17 | 0.0213 |
| **SynDST11** | 207 | 6 | 0.0075 | 206 | 6 | 0.0075 | 214 | 12 | 0.0150 |
| **SynDST12** | 186 | 8 | 0.0100 | 187 | 9 | 0.0113 | 200 | 15 | 0.0188 |
| **Average** | 168.5 | 6.1 | **0.0076** | 182.7 | 7.9 | **0.0099** | 189.2 | 14.3 | **0.0178** |

**Supplementary Table 2.** Performance comparison between DCPR and baselines on twelve time-course synthetic datasets

|  |  | **AUC** | **MedAE (h)** | **SDAE (h)** | **CCC** | **Percent within 1h** | **Percent within 2h** |
| --- | --- | --- | --- | --- | --- | --- | --- |
| **SynDST1** | DCPR | 1.000 | 0.042 | 0.006 | 1.000 | 100 | 100 |
|  | CYCLOPS | 0.624 | 2.876 | 4.072 | 0.052 | 16.67 | 33.33 |
|  | CHIRAL | 0.980 | 0.272 | 0.308 | 0.985 | 95.83 | 100 |
|  | Cyclum | 0.952 | 0.511 | 0.499 | 0.960 | 62.50 | 100 |
| **SynDST2** | DCPR | 0.911 | 0.029 | 1.949 | 0.746 | 66.67 | 77.78 |
|  | CYCLOPS | 0.690 | 2.176 | 3.244 | 0.206 | 11.11 | 27.78 |
|  | CHIRAL | 0.901 | 0.691 | 1.219 | 0.806 | 66.67 | 72.22 |
|  | Cyclum | 0.887 | 0.514 | 2.506 | 0.623 | 72.22 | 83.33 |
| **SynDST3** | DCPR | 0.948 | 0.597 | 0.565 | 0.949 | 75 | 95 |
|  | CYCLOPS | 0.734 | 2.142 | 3.312 | 0.280 | 30 | 50 |
|  | CHIRAL | 0.944 | 0.658 | 0.734 | 0.934 | 80 | 90 |
|  | Cyclum | 0.942 | 0.295 | 1.112 | 0.896 | 75 | 90 |
| **SynDST4** | DCPR | 0.954 | 0.451 | 0.471 | 0.958 | 70.83 | 100 |
|  | CYCLOPS | 0.565 | 3.543 | 4.109 | 0.057 | 8.33 | 25 |
|  | CHIRAL | 0.960 | 0.436 | 0.457 | 0.966 | 79.17 | 100 |
|  | Cyclum | 0.961 | 0.488 | 0.379 | 0.975 | 79.17 | 100 |
| **SynDST5** | DCPR | 0.994 | 0.032 | 0.183 | 0.997 | 100 | 100 |
|  | CYCLOPS | 0.636 | 3.278 | 3.106 | 0.079 | 6.25 | 18.75 |
|  | CHIRAL | 0.971 | 0.323 | 0.377 | 0.977 | 89.58 | 100 |
|  | Cyclum | 0.968 | 0.352 | 0.432 | 0.973 | 87.50 | 97.92 |
| **SynDST6** | DCPR | 1.000 | 0.036 | 0.009 | 1.000 | 100 | 100 |
|  | CYCLOPS | 0.731 | 2.516 | 3.085 | 0.285 | 25 | 41.67 |
|  | CHIRAL | 0.967 | 0.407 | 0.368 | 0.976 | 85.42 | 100 |
|  | Cyclum | 0.957 | 0.404 | 0.583 | 0.956 | 77.08 | 97.92 |
| **SynDST7** | DCPR | 1.000 | 0.034 | 0.017 | 1.000 | 100 | 100 |
|  | CYCLOPS | 0.766 | 1.689 | 2.747 | 0.350 | 22.22 | 55.56 |
|  | CHIRAL | 0.938 | 0.440 | 1.027 | 0.905 | 75 | 86.11 |
|  | Cyclum | 0.919 | 0.431 | 1.802 | 0.794 | 72.22 | 86.11 |
| **SynDST8** | DCPR | 0.963 | 0.039 | 0.823 | 0.942 | 80.95 | 90.48 |
|  | CYCLOPS | 0.789 | 1.374 | 3.030 | 0.399 | 33.33 | 59.52 |
|  | CHIRAL | 0.927 | 0.423 | 1.712 | 0.795 | 71.43 | 88.10 |
|  | Cyclum | 0.900 | 0.661 | 2.200 | 0.742 | 64.29 | 90.48 |
| **SynDST9** | DCPR | 0.952 | 0.574 | 0.622 | 0.956 | 75 | 95.83 |
|  | CYCLOPS | 0.651 | 2.733 | 3.459 | 0.119 | 10.42 | 29.17 |
|  | CHIRAL | 0.974 | 0.338 | 0.316 | 0.978 | 95.83 | 100 |
|  | Cyclum | 0.905 | 0.966 | 1.105 | 0.842 | 50 | 83.33 |
| **SynDST10** | DCPR | 0.802 | 2.142 | 1.843 | 0.478 | 28.57 | 42.86 |
|  | CYCLOPS | 0.587 | 4.238 | 3.467 | 0.052 | 14.29 | 21.43 |
|  | CHIRAL | 0.681 | 2.234 | 3.748 | 0.088 | 21.43 | 35.71 |
|  | Cyclum | 0.672 | 3.232 | 2.607 | 0.124 | 0.00 | 14.29 |
| **SynDST11** | DCPR | 0.862 | 1.422 | 1.486 | 0.717 | 33.33 | 66.67 |
|  | CYCLOPS | 0.822 | 1.176 | 3.470 | 0.442 | 33.33 | 61.11 |
|  | CHIRAL | 0.833 | 1.075 | 2.817 | 0.281 | 44.44 | 66.67 |
|  | Cyclum | 0.852 | 1.261 | 1.298 | 0.798 | 22.22 | 61.11 |
| **SynDST12** | DCPR | 0.858 | 1.344 | 1.302 | 0.813 | 33.33 | 55.56 |
|  | CYCLOPS | 0.807 | 2.014 | 1.873 | 0.337 | 22.22 | 44.44 |
|  | CHIRAL | 0.810 | 1.860 | 2.172 | 0.453 | 27.78 | 55.56 |
|  | Cyclum | 0.615 | 4.100 | 3.385 | 0.049 | 16.67 | 22.22 |

**Supplementary Table 3.** Performance (AUCs) comparison between DCPR and baseline methods on three time-course GEO datasets. In the training process of DCPR, homologous genes are selected using JTK_CYCLE model (*p* < 0.01).

|  |  | **GSE223761** | **GSE161566** | **GSE56931** |
| --- | --- | --- | --- | --- |
| CYCLOPS | Seed genes | 0.834 | 0.662 | 0.711 |
| CHIRAL | Seed genes | 0.763 | 0.722 | 0.737 |
| Cyclum | Seed genes | 0.745 | 0.693 | 0.771 |
| DCPR | Seed genes | **0.897** | **0.831** | **0.803** |
| CYCLOPS | All genes | 0.589 | 0.593 | 0.728 |
| CHIRAL | All genes | 0.712 | 0.694 | 0.751 |
| Cyclum | All genes | 0.746 | 0.767 | 0.781 |
| DCPR | All genes | **0.809** | **0.771** | **0.797** |

**Supplementary Table 4.** Prior knowledge items consistent with the prediction results of all four models on GSE11923

| **Error range** | **DCPR** | **CYCLOPS** | **CHIRAL** | **Cyclum** |
| --- | --- | --- | --- | --- |
| $\pm$1 hour | 730 | 545 | 549 | 579 |
| $\pm$2 hour | 1,785 | 1,448 | 1,382 | 1,438 |

**Supplementary Table 5**. Performance comparison between DCPR and baseline methods on GSE11923. The prior knowledge associated with all clock genes established through experimental validation, is collected from CGDB database. The mean absolute error of phase estimation is present if a gene expression series exhibits significant rhythmicity (*p* < 0.05).

| **Gene** | **Phase** | **Peak** | **Trough** | **PMID** | **Peak_DP_** | **Trough_DP_** | **pvalue_DP_** | **Err_DP_** | **Err_CP_** | **Err_CM_** | **Err_CR_** |
| --- | --- | --- | --- | --- | --- | --- | --- | --- | --- | --- | --- |
| *Arntl* | CT 23 11 | 23 | 11 | 24591654 | 22.73 | 10.71 | 2.22E-16 | **0.28** | - | 1.41 | 0.66 |
| *Bhlhe41* | CT 11 23 | 11 | 23 | 23217262 | 14.89 | 2.93 | 1.19E-02 | **3.91** | 8.43 | - | 6.03 |
| *Clock* | CT 0 12 | 0 | 12 | 11779462  23217262 | 23.30 | 11.29 | 1.44E-15 | 0.70 | - | **0.03** | 0.94 |
| *Cry1* | CT 20 8 | 21 | 9 | 24591654 | 20.85 | 8.84 | 3.05E-05 | **0.15** | 1.83 | 2.32 | - |
| *Cry2* | CT 10 22 | 9 | 21 | 11779462 | 9.32 | 21.33 | 4.13E-05 | **0.33** | - | - | - |
| *Dbp* | CT 9 21 | 9 | 21 | 11316793 | 9.66 | 21.62 | 1.81E-10 | 0.64 | - | - | **0.42** |
| *Fbxo21* | CT 6 20 | 6 | 20 | 19343201 | 6.58 | 18.55 | 1.29E-07 | **1.02** | 1.71 | 1.23 | - |
| *Fbxo3* | CT 20 7 | 20 | 7 | 19343201 | 21.29 | 9.27 | 1.73E-02 | **1.78** | - | - | 1.78 |
| *Hlf* | CT 11 23 | 11 | 23 | 23217262 | 9.90 | 21.86 | 1.93E-07 | **1.12** | - | - | 1.53 |
| *Nampt* | CT 14 3 | 14 | 3 | 19343201 | 13.93 | 1.92 | 8.79E-12 | **0.57** | 0.82 | 0.91 | 1.73 |
| *Nfil3* | CT 23 11 | 23 | 11 | 23217262  24591654 | 23.26 | 11.29 | 7.48E-10 | **0.27** | - | 1.29 | 0.30 |
| *Npas2* | CT 2 8 | 2 | 8 | 19343201 | 0.29 | 12.30 | 3.59E-04 | **3.01** | - | 3.01 | - |
| *Nr1d1* | CT 4 16 | 4 | 16 | 20937769 | 6.34 | 18.35 | 1.86E-05 | 2.35 | **0.22** | 0.62 | - |
| *Nr1d2* | CT 8 0 | 8 | 0 | 20937769 | 9.66 | 21.67 | 3.44E-13 | **1.99** | - | - | - |
| *Per1* | CT 12 0 | 12 | 0 | 20937769 | 11.15 | 23.16 | 6.67E-06 | **0.85** | - | - | - |
| *Per2* | CT 15 3 | 15 | 3 | 9655499 | 13.26 | 1.25 | 9.51E-09 | 1.74 | 0.16 | **0.06** | 3.01 |
| *Per3* | CT 15 3 | 15 | 3 | 9655499 | 9.85 | 21.86 | 4.43E-05 | **5.14** | - | - | - |
| *Rora* | CT 20 14 | 20 | 14 | 19343201 | - | - | 1.43E-01 | **-** | - | 3.01 | **2.98** |
| *Rorc* | CT 18 6 | 18 | 6 | 23217262 | 19.65 | 7.64 | 5.15E-03 | 1.65 | 1.26 | **0.13** | - |
| *Tef* | CT 12 0 | 12 | 0 | 11316793  23217262  24591654 | 10.52 | 22.53 | 1.27E-14 | **1.47** | - | - | 2.24 |

**Supplementary Table 6**. Performance comparison of three baselines on GSE11923. The information of peak and trough are presented if a gene expression series exhibits significant rhythmicity (*p* < 0.05).

| **Gene** | **Phase** | **Peak_CP_** | **Trough_CP_** | **pvalue_CP_** | **Peak_CM_** | **Trough_CM_** | **pvalue_CM_** | **Peak_CR_** | **Trough_CR_** | **pvalue_CR_** |
| --- | --- | --- | --- | --- | --- | --- | --- | --- | --- | --- |
| *Arntl* | CT 23 11 | - | - | 1.87E-01 | 21.57 | 9.61 | 3.31E-02 | 22.34 | 10.33 | 3.66E-02 |
| *Bhlhe41* | CT 11 23 | 19.41 | 7.45 | 1.91E-02 | - | - | 7.08E-01 | 17.01 | 5.05 | 4.22E-03 |
| *Clock* | CT 0 12 | - | - | 1.41E-01 | 23.98 | 11.96 | 4.02E-02 | 23.06 | 11.05 | 8.45E-04 |
| *Cry1* | CT 21 9 | 19.17 | 7.16 | 2.33E-02 | 18.69 | 6.68 | 6.96E-04 | - | - | 1.08E-01 |
| *Cry2* | CT 9 21 | - | - | 1.07E-01 | - | - | 1.44E-01 | - | - | 3.90E-01 |
| *Dbp* | CT 9 21 | - | - | 5.13E-02 | - | - | 2.57E-01 | 9.42 | 21.43 | 4.39E-02 |
| *Fbxo21* | CT 6 20 | 5.29 | 17.30 | 9.85E-05 | 5.77 | 17.78 | 1.56E-02 | - | - | 2.38E-01 |
| *Fbxo3* | CT 20 7 | - | - | 7.19E-02 | - | - | 7.33E-02 | 21.29 | 9.27 | 2.42E-03 |
| *Hlf* | CT 11 23 | - | - | 7.22E-02 | - | - | 9.46E-01 | 9.47 | 21.48 | 3.32E-02 |
| *Nampt* | CT 14 3 | 15.33 | 3.32 | 4.10E-07 | 13.60 | 1.59 | 7.04E-03 | 12.78 | 0.77 | 3.34E-06 |
| *Nfil3* | CT 23 11 | - | - | 3.78E-01 | 21.72 | 9.71 | 6.81E-03 | 23.30 | 11.29 | 5.41E-03 |
| *Npas2* | CT 2 8 | - | - | 2.62E-01 | 0.67 | 12.68 | 5.73E-03 |  |  | 6.53E-02 |
| *Nr1d1* | CT 4 16 | 3.80 | 15.76 | 9.16E-04 | 4.61 | 16.62 | 9.59E-04 | - | - | 1.90E-01 |
| *Nr1d2* | CT 8 0 | - | - | 6.78E-02 |  |  | 8.94E-02 | - | - | 1.24E-01 |
| *Per1* | CT 12 0 | - | - | 1.48E-01 | **-** | - | 3.19E-01 | - | - | 1.31E-01 |
| *Per2* | CT 15 3 | 14.85 | 2.83 | 2.35E-04 | 15.04 | 3.08 | 2.10E-02 | 12.01 | 23.98 | 7.23E-04 |
| *Per3* | CT 15 3 | - | - | 2.07E-01 | - | - | 8.60E-01 | - | - | 1.02E-01 |
| *Rora* | CT 20 14 | - | - | 1.45E-01 | 20.47 | 8.46 | 2.87E-03 | 23.69 | 11.72 | 1.36E-02 |
| *Rorc* | CT 18 6 | 19.27 | 7.26 | 1.25E-02 | 17.87 | 5.86 | 1.79E-06 | - | - | 1.40E-01 |
| *Tef* | CT 12 0 | - | - | 1.28E-01 | - | - | 2.52E-01 | 9.75 | 21.77 | 1.91E-02 |

**Supplementary Table 7**. Clock genes in AD with altered rhythms identified based on DCPR-predicted phase

| **Brain Region** | **Clock gene** | **Control** | **AD** | **Difference type** | **Diff-pval** |
| --- | --- | --- | --- | --- | --- |
| EC | *CLOCK* | 4.32E-02 | 1.65E-01 | Loss of rhythmicity |  |
|  | *ARNTL* | 3.32E-02 | 8.58E-01 | Loss of rhythmicity |  |
|  | *CRY1* | 3.73E-02 | 1.63E-01 | Loss of rhythmicity |  |
|  | *CRY2* | 2.88E-02 | 4.16E-01 | Loss of rhythmicity |  |
|  | *DBP* | 2.66E-03 | 6.89E-01 | Loss of rhythmicity |  |
|  | *NPAS2* | 1.79E-02 | 2.02E-01 | Loss of rhythmicity |  |
|  | *TEF* | 4.98E-02 | 7.24E-01 | Loss of rhythmicity |  |
|  | *HLF* | 2.53E-03 | 6.79E-01 | Loss of rhythmicity |  |
| HIP | *CLOCK* | 1.65E-05 | 7.98E-03 | Period change |  |
|  | *ARNTL* | 4.61E-04 | 8.70E-03 | Phase shift | 4.74E-03 |
|  | *ARNTL2* | 1.11E-02 | 6.48E-02 | Loss of rhythmicity |  |
|  | *CRY1* | 7.88E-03 | 4.68E-01 | Loss of rhythmicity |  |
|  | *CRY2* | 4.05E-02 | 2.71E-01 | Loss of rhythmicity |  |
|  | *DEC1* | 1.64E-01 | 1.83E-03 | Gain of rhythmicity |  |
|  | *DEC2* | 9.36E-03 | 1.25E-01 | Loss of rhythmicity |  |
|  | *RORA* | 7.16E-04 | 2.06E-02 | Period change |  |
|  | *RORB* | 1.94E-02 | 6.50E-02 | Loss of rhythmicity |  |
|  | *PER1* | 3.48E-04 | 2.93E-03 | Period change |  |
|  | *PER3* | 4.58E-02 | 3.45E-04 | Period change |  |
|  | *DBP* | 1.59E-03 | 7.06E-03 | Period change |  |
|  | *NR1D2* | 3.88E-02 | 1.88E-01 | Loss of rhythmicity |  |
|  | *NPAS2* | 3.71E-03 | 1.15E-03 | Period change |  |
|  | *HLF* | 2.43E-02 | 1.84E-02 | Period change |  |
| FC | *CLOCK* | 2.83E-03 | 3.85E-01 | Loss of rhythmicity |  |
|  | *ARNTL* | 1.51E-02 | 9.95E-02 | Loss of rhythmicity |  |
|  | *CRY1* | 1.47E-04 | 2.32E-01 | Loss of rhythmicity |  |
|  | *CRY2* | 2.38E-04 | 2.90E-01 | Loss of rhythmicity |  |
|  | *DEC2* | 2.68E-03 | 8.21E-01 | Loss of rhythmicity |  |
|  | *RORB* | 1.39E-02 | 3.68E-02 | Phase shift  Base shift | 2.17E-02  6.13E-03 |
|  | *DBP* | 8.63E-04 | 4.20E-01 | Loss of rhythmicity |  |
|  | *NR1D1* | 4.65E-02 | 3.38E-01 | Loss of rhythmicity |  |
|  | *TEF* | 2.46E-02 | 8.05E-01 | Loss of rhythmicity |  |
|  | *HLF* | 1.10E-06 | 3.70E-02 | Amplitude change  Phase shift  Base shift | 5.27E-03  1.00E-02  1.65E-03 |

**Supplementary Table 8.** Performance of DCPR and its variants on six GEO datasets

| **Datasets** | **AUC** | | | | **MedAE** | | | |
| --- | --- | --- | --- | --- | --- | --- | --- | --- |
|  | DCPR ($M_{0}$) | $M_{1}$ | $M_{2}$ | $M_{3}$ | DCPR ($M_{0}$) | $M_{1}$ | $M_{2}$ | $M_{3}$ |
| GSE223761 | **0.897** | 0.891 | 0.789 | 0.773 | **1.179** | 1.190 | 2.000 | 2.003 |
| GSE161566 | **0.831** | 0.827 | 0.806 | 0.824 | **0.861** | 1.252 | 1.696 | 1.252 |
| GSE56931 | **0.803** | 0.787 | 0.740 | 0.712 | **1.442** | 1.525 | 2.164 | 1.954 |
| GSE11923 | **0.828** | 0.797 | 0.769 | 0.738 | **1.018** | 1.741 | 1.792 | 1.922 |
| GSE50438 | **0.840** | 0.745 | 0.740 | 0.693 | **1.504** | 1.927 | 1.660 | 2.642 |
| GSE259431 | **0.846** | 0.842 | 0.808 | 0.749 | 1.079 | **1.070** | 1.797 | 2.370 |

**Supplementary Table 9.** Impact of DCPR-Derived Preprocessing (DP_pre) on the Performance of SOTA Models

|  |  | **AUC** | **MedAE (h)** | **SDAE (h)** | **CCC** | **Percent within 1h** | **Percent within 2h** |
| --- | --- | --- | --- | --- | --- | --- | --- |
| **GSE223761** | DCPR | 0.897 | 1.179 | 0.840 | 0.879 | 50.00 | 83.33 |
|  | CYCLOPS | 0.834 | 1.366 | 1.488 | 0.629 | 0 | 83.33 |
|  | CYCLOPS + DP_pre | 0.843 | 1.008 | 2.366 | 0.562 | 50.00 | 70.00 |
|  | CHIRAL | 0.763 | 1.737 | 2.055 | 0.727 | 0 | 50.00 |
|  | CHIRAL + DP_pre | 0.833 | 1.930 | 1.341 | 0.623 | 16.67 | 50.00 |
|  | Cyclum | 0.745 | 2.301 | 2.197 | 0.355 | 0 | 50.00 |
|  | Cyclum + DP_pre | 0.809 | 2.000 | 2.213 | 0.458 | 16.67 | 50.00 |
| **GSE161566** | DCPR | 0.831 | 0.861 | 2.204 | 0.631 | 41.67 | 75.00 |
|  | CYCLOPS | 0.662 | 2.806 | 3.471 | 0.143 | 14.58 | 33.33 |
|  | CYCLOPS + DP_pre | 0.734 | 2.142 | 3.312 | 0.280 | 30.00 | 50.00 |
|  | CHIRAL | 0.722 | 2.997 | 3.335 | 0.138 | 33.33 | 54.17 |
|  | CHIRAL + DP_pre | 0.781 | 1.831 | 1.979 | 0.493 | 8.33 | 58.33 |
|  | Cyclum | 0.693 | 2.384 | 3.098 | 0.199 | 10.00 | 35.00 |
|  | Cyclum + DP_pre | 0.806 | 1.805 | 2.109 | 0.539 | 25.00 | 58.33 |
| **GSE56931** | DCPR | 0.803 | 1.442 | 2.331 | 0.481 | 33.33 | 64.81 |
|  | CYCLOPS | 0.711 | 2.184 | 2.874 | 0.014 | 12.50 | 45.83 |
|  | CYCLOPS + DP_pre | 0.727 | 1.932 | 3.335 | 0.138 | 33.33 | 54.17 |
|  | CHIRAL | 0.737 | 2.163 | 2.761 | 0.302 | 14.58 | 41.67 |
|  | CHIRAL + DP_pre | 0.738 | 2.251 | 2.680 | 0.316 | 15.42 | 43.21 |
|  | Cyclum | 0.771 | 1.634 | 3.170 | 0.360 | 35.42 | 60.42 |
|  | Cyclum + DP_pre | 0.799 | 0.830 | 3.615 | 0.331 | 55.56 | 72.22 |
| **GSE11923** | DCPR | 0.828 | 1.018 | 2.449 | 0.542 | 47.92 | 62.50 |
|  | CYCLOPS | 0.724 | 1.771 | 3.220 | 0.239 | 25.00 | 52.08 |
|  | CYCLOPS + DP_pre | 0.742 | 2.075 | 2.600 | 0.306 | 12.50 | 47.92 |
|  | CHIRAL | 0.683 | 2.999 | 2.926 | 0.154 | 16.67 | 41.67 |
|  | CHIRAL + DP_pre | 0.782 | 1.665 | 2.848 | 0.432 | 29.17 | 54.17 |
|  | Cyclum | 0.704 | 3.063 | 2.887 | 0.250 | 20.83 | 37.50 |
|  | Cyclum + DP_pre | 0.713 | 2.968 | 2.573 | 0.255 | 20.83 | 33.33 |
| **GSE50438** | DCPR | 0.840 | 1.504 | 1.952 | 0.641 | 43.06 | 66.67 |
|  | CYCLOPS | 0.812 | 1.214 | 2.282 | 0.541 | 47.22 | 59.72 |
|  | CYCLOPS + DP_pre | 0.833 | 1.930 | 1.341 | 0.623 | 16.67 | 50.00 |
|  | CHIRAL | 0.820 | 1.584 | 1.705 | 0.646 | 20.83 | 68.06 |
|  | CHIRAL + DP_pre | 0.827 | 1.637 | 2.342 | 0.561 | 28.33 | 58.75 |
|  | Cyclum | 0.685 | 2.149 | 3.585 | 0.174 | 25.00 | 50.00 |
|  | Cyclum + DP_pre | 0.712 | 2.207 | 2.874 | 0.014 | 12.50 | 45.83 |
| **GSE259431** | DCPR | 0.846 | 1.079 | 1.952 | 0.636 | 47.92 | 64.58 |
|  | CYCLOPS | 0.753 | 1.815 | 2.722 | 0.252 | 25.00 | 55.21 |
|  | CYCLOPS + DP_pre | 0.789 | 1.374 | 3.030 | 0.399 | 33.33 | 59.52 |
|  | CHIRAL | 0.663 | 3.034 | 3.180 | 0.153 | 19.79 | 32.29 |
|  | CHIRAL + DP_pre | 0.685 | 2.377 | 3.635 | 0.102 | 33.33 | 45.83 |
|  | Cyclum | 0.805 | 1.268 | 2.546 | 0.502 | 33.33 | 66.67 |
|  | Cyclum + DP_pre | 0.839 | 0.910 | 2.634 | 0.542 | 52.08 | 66.67 |

**Supplementary Table 10.** DCPR’s performance on two datasets reported by other algorithms

|  |  | **AUC** | **MedAE (h)** | **SDAE (h)** | **CCC** | **Percent within 1h** | **Percent within 2h** |
| --- | --- | --- | --- | --- | --- | --- | --- |
| **GSE71620** | DCPR | **0.709** | 2.290 | **2.701** | **0.217** | **16.22** | **43.24** |
|  | CYCLOPS | 0.550 | 4.732 | 3.248 | 0.018 | 5.88 | 16.18 |
|  | CHIRAL | 0.701 | **2.219** | 3.164 | 0.076 | 10.29 | 38.24 |
|  | Cyclum | 0.578 | 4.258 | 3.471 | 0.029 | 11.76 | 23.53 |
| **GSE108539** | DCPR | 0.833 | **1.113** | 2.815 | 0.540 | **46.43** | **64.29** |
|  | CYCLOPS | 0.581 | 4.138 | 3.255 | 0.015 | 12.28 | 19.30 |
|  | CHIRAL | **0.849** | 1.156 | **1.862** | **0.646** | 43.86 | 63.16 |
|  | Cyclum | 0.624 | 3.707 | 3.253 | 0.057 | 8.77 | 26.32 |

**Supplementary Table 11.** DCPR’s Performance on GSE196430 dataset from Bmal1 knockout mice. Samples from wide type (WT) and Bmal1 knock out (KO) are tested.

|  |  | **AUC** | **MedAE (h)** | **SDAE (h)** | **CCC** | **Percent within 1h** | **Percent within 2h** |
| --- | --- | --- | --- | --- | --- | --- | --- |
| **GSE196430**  **(WT)** | DCPR | **0.940** | **0.417** | 1.006 | 0.913 | 72.22 | 83.33 |
|  | CYCLOPS | 0.931 | 0.703 | **0.606** | **0.949** | 61.11 | 94.44 |
|  | CHIRAL | 0.937 | 0.541 | 0.610 | 0.931 | 66.67 | 88.89 |
|  | Cyclum | 0.917 | 0.760 | 0.699 | 0.911 | 61.11 | 88.89 |
| **GSE196430（KO）** | DCPR | **0.764** | 2.012 | **2.544** | 0.345 | 27.78 | 44.44 |
|  | CYCLOPS | 0.673 | 2.533 | 3.616 | 0.146 | 16.67 | 33.33 |
|  | CHIRAL | 0.751 | **1.967** | 3.395 | **0.392** | 33.33 | 50.00 |
|  | Cyclum | 0.631 | 2.539 | 4.008 | 0.004 | 22.22 | 38.89 |

**Supplementary Table 12.** DCPR’s Performance on GSE135898 dataset from Cry1/2 KO and Bmal1 KO under Ad Libitum (AL) and Night Restricted-feeding (NRF) Regimens

| **Datasets** | **Methods** | **AUC** | **MedAE (h)** | **SDAE (h)** | **CCC** | **Percent within 1h** | **Percent within 2h** |
| --- | --- | --- | --- | --- | --- | --- | --- |
| GSE135898  (AL_Cry1/2 WT) | DCPR | **1.000** | **0.027** | 0.014 | 1.000 | 100.00 | 100.00 |
|  | CYCLOPS | 0.858 | 0.635 | 2.192 | 0.542 | 66.67 | 66.67 |
|  | CHIRAL | 0.924 | 0.650 | 0.978 | 0.936 | 66.67 | 83.33 |
|  | Cyclum | 0.768 | 2.104 | 2.315 | 0.392 | 8.33 | 50.00 |
| GSE135898  (AL_Cry1/2 KO) | DCPR | **0.802** | **1.498** | 2.573 | 0.319 | 33.33 | 58.33 |
|  | CYCLOPS | 0.548 | 5.331 | 3.610 | 0.000 | 8.33 | 16.67 |
|  | CHIRAL | 0.643 | 3.281 | 3.535 | 0.055 | 16.67 | 16.67 |
|  | Cyclum | 0.785 | 1.883 | 2.140 | 0.413 | 25.00 | 58.33 |
| GSE135898  (AL_Bmal1 WT) | DCPR | 0.865 | 0.743 | 2.089 | 0.582 | 50.00 | 66.67 |
|  | CYCLOPS | 0.736 | 2.410 | 2.894 | 0.043 | 25.00 | 33.33 |
|  | CHIRAL | **0.913** | **0.623** | 1.092 | 0.867 | 66.67 | 66.67 |
|  | Cyclum | 0.819 | 1.353 | 2.687 | 0.291 | 33.33 | 75.00 |
| GSE135898  (AL_Bmal1 KO) | DCPR | **0.814** | **1.974** | 2.210 | 0.417 | 33.33 | 58.33 |
|  | CYCLOPS | 0.695 | 2.416 | 3.471 | 0.067 | 8.33 | 50.00 |
|  | CHIRAL | 0.726 | 2.534 | 3.156 | 0.245 | 25.00 | 25.00 |
|  | Cyclum | 0.703 | 2.566 | 3.210 | 0.094 | 25.00 | 33.33 |
| GSE135898  (NRF_Cry1/2 WT) | DCPR | **0.868** | **1.007** | 2.783 | 0.602 | 50.00 | 75.00 |
|  | CYCLOPS | 0.756 | 1.980 | 1.796 | 0.431 | 0.00 | 58.33 |
|  | CHIRAL | 0.838 | 1.503 | 2.239 | 0.698 | 50.00 | 50.00 |
|  | Cyclum | 0.823 | 1.500 | 2.300 | 0.390 | 33.33 | 58.33 |
| GSE135898  (NRF_Cry1/2 KO) | DCPR | **0.807** | **0.438** | 3.486 | 0.341 | 58.33 | 66.67 |
|  | CYCLOPS | 0.546 | 5.106 | 3.580 | 0.002 | 16.67 | 16.67 |
|  | CHIRAL | 0.698 | 2.221 | 3.124 | 0.249 | 8.33 | 25.00 |
|  | Cyclum | 0.782 | 1.311 | 3.476 | 0.157 | 50.00 | 58.33 |
| GSE135898  (NRF_Bmal1 WT) | DCPR | **0.846** | **0.212** | 2.578 | 0.488 | 58.33 | 58.33 |
|  | CYCLOPS | 0.575 | 4.337 | 3.838 | 0.029 | 16.67 | 25.00 |
|  | CHIRAL | 0.838 | 1.377 | 1.803 | 0.690 | 33.33 | 58.33 |
|  | Cyclum | 0.820 | 1.568 | 2.147 | 0.489 | 41.67 | 58.33 |
| GSE135898  (NRF_Bmal1 KO) | DCPR | **0.804** | 1.866 | 1.928 | 0.530 | 33.33 | 58.33 |
|  | CYCLOPS | 0.608 | 4.283 | 3.099 | 0.042 | 8.33 | 16.67 |
|  | CHIRAL | 0.722 | **0.268** | 2.093 | 2.977 | 8.33 | 50.00 |
|  | Cyclum | 0.749 | 1.589 | 3.514 | 0.285 | 16.67 | 58.33 |

**Supplementary Table 13.** Hyperparameter setting for DCPR model

| **Hyperparameter** | **Value** |
| --- | --- |
| Batch size | 1 |
| Attention hidden size | 6 |
| Attention heads | 2 |
| Learning rate | 0.0001 |
| Optimizer | Adam |
| Maximal epoch | 10000 |

**Supplementary Table 14.** Default setting of key parameters and statistical thresholds reported in SOTA models

| **Methods** | **Key parameters** | **Default value or threshold** | **Version** |
| --- | --- | --- | --- |
| CHIRAL | Prior probability of rhythmic genes  Maximum iterations for EM algorithm  Row-wise mean centering  Maintain original variance of gene expression  Core clock gene set (uses all genes if NULL) | q = 0.1  iterations >= 500  mean.centre.E = TRUE  standardize = FALSE  **clockgenes = NULL** | R |
| CYCLOPS | **Threshold for rhythmic gene significance (uses all genes if no homology)**  Number of top-expressed genes retained  Variance of the data explained threshold (SVD) | q < 0.05  n_gene_kept = 10000  variance_keptt = 0.85 | Python  (Code is provided by Cyclum) |
| Cyclum | Number of circular components  Number of linear bypass components  Encoder hidden layers | n_circular_unit = 1  n_linear_bypass = 0  encoder_depth = 2 | Python |
